# Synergistic Antibacterial Potential and Cell Surface Topology Study of Carbon Nanodots and Tetracycline Against *E. coli*

**DOI:** 10.3389/fbioe.2021.626276

**Published:** 2021-10-05

**Authors:** Dhermendra K. Tiwari, Gargi Jha, Manisha Tiwari, Savita Kerkar, Suman Das, Vivekanand V. Gobre

**Affiliations:** ^1^ Department of Biotechnology, Faculty of Life Sciences and Environment, Goa University, Taleigao plateau, Goa, India; ^2^ School of Chemical Sciences, Goa University, Taleigao plateau, Goa, India

**Keywords:** carbon dot, antibacterial, carbon nanomaterial, synergistic, atomic force microscopy, antibiotic resistance

## Abstract

Increasing drugs and antibiotic resistance against pathogenic bacteria create the necessity to explore novel biocompatible antibacterial materials. This study investigated the antibacterial effect of carbon dot (C-dot) against *E. coli* and suggested an effective synergistic dose of tetracycline with C-dot, using mathematical modeling of antibacterial data. Colony count and growth curve studies clearly show an enhanced antibacterial activity against *E. coli* synergistically treated with C-dot and tetracycline, even at a concentration ten times lower than the minimum inhibitory concentration (MIC). The Richards model-fit of growth curve clearly showed an increase in doubling time, reduction in growth rate, and early stationary phase in the synergistic treatment with 42% reduction in the growth rate (μ_m_) compared to the control. Morphological studies of *E. coli* synergistically treated with C-dot + tetracycline showed cell damage and deposition of C-dots on the bacterial cell membrane in scanning electron microscopy imaging. We further validated the topological changes, cell surface roughness, and significant changes in the height profile (ΔZ) with the control and treated *E. coli* cells viewed under an atomic force microscope. We confirmed that the effective antibacterial doses of C-dot and tetracycline were much lower than the MIC in a synergistic treatment.

## Introduction

Several studies suggest that nanoparticles (NPs) can be an excellent replacement to antibiotics and effective drug carriers and are suitable for multifunctional designs with their broad-spectrum antibacterial properties, considering the availability of various functional groups (Mühling et al., 2009; Pelgrift and Friedman, 2013; Beyth et al., 2015). Nanoparticles not only combat antibiotic resistance but also act as a carrier of drugs/antibiotics such as liposomal NPs (Daeihamed et al., 2016) and solid lipid (SL) NPs (Thukral et al., 2014; Naseri et al., 2015) used in drug delivery and a wide range of therapeutics (Pissuwan et al., 2011; Gholipourmalekabadi et al., 2017). Carbon nanodots (C-dots) or carbon quantum dots are one of the most recently discovered and effective antimicrobial agents (Thakur et al., 2014; Lim et al., 2015; Maas, 2016; Meziani et al., 2016; Yang et al., 2016; Dong et al., 2017; Xin et al., 2019; Muktha et al., 2020). They are carbon-based nanoparticles with a size less than 10 nm and have surface passivation properties (Sun et al., 2006; LeCroy et al., 2016). C-dot possesses photo excited state properties and redox processes similar to conventional nanoscale semiconductors (Rocha et al., 2002; Hu et al., 2015; Jelinek, 2017; Righetto et al., 2017), which initiate the efficient photoinduced charge separation. This process allows the formation of radical anions and cations (Cao et al., 2013; Fernando et al., 2015; LeCroy et al., 2016). These photo-generated electrons and holes in carbon dots are the basis of catalytic processes (Cao et al., 2013; Fernando et al., 2015), providing a strong photodynamic effect to C-dots (Luo et al., 2014). Due to their photoinduced redox potential, C-dots are excellent candidates functioning as antibacterial agents and are effective against bacterial cells, particularly under normal visible/natural light illumination without giving an excess dose of the photon (Lim et al., 2015; Meziani et al., 2016). An excess amount of photons also triggers C-dot–dependent photodamage of tetracycline and other similar antibiotics (López-Peñalver et al., 2010; Nagamine et al., 2020; Semeraro et al., 2020; Xu et al., 2020; Liu et al., 2021).

When traditional antibacterial reagents are compared with C-dot, the latter proves to be advantageous as they are intrinsically non-toxic both *in vitro* and *in vivo* (Najjar et al., 2007; Li et al., 2014a) and environment-friendly, whereas antibacterial chemicals mostly exhibit potential toxicity to the environment and ecological elements. As discussed earlier, the unchecked use of antibiotics and other antibacterial substances has led to the emergence of multidrug-resistant bacteria (MDR). Thus, the C-dot being non-toxic and environment-friendly can be effectively used against these MDR bacteria and helps control infections in public health sectors. Studies have suggested a direct interaction between carbon-based nanoparticles with various bacterial and fungal cells (Zou et al., 2016). Studies on the antimicrobial activities of carbon dots are increasing with time, and several studies suggested mechanisms that include oxidative stress (Akhavan et al., 2011; Li et al., 2014b). Currently, few reports are available on the potential of carbon dots having an antimicrobial effect on *E. coli* and other microorganisms (Thakur et al., 2014; Meziani et al., 2016; Dong et al., 2018; Dong et al., Elcodous et al., 2020; 2020; Mukhta et al., 2020).

C-dots obtained from gum arabic showed potential antimicrobial activity against Gram-positive and Gram-negative bacteria (Thukral et al., 2014). Nanocarbons synthesized by green synthesis from turmeric smudge, pomegranate peel, and watermelon peel have also been reported with potent antibacterial activity against *E. coli*, *Bacillus subtilis*, and *Pseudomonas aeruginosa* (Chun et al., 2016; Muktha et al., 2020). In recent years, even though many studies have emerged on the use of C-dots for various applications, more systematic studies on the antimicrobial property are required to understand the exact mechanisms of their actions. Detailed analysis of the antibacterial effect and the mathematical modeling of the obtained data are necessary to understand the minute changes to identify the effective doses of such broad-spectrum antibacterial agents (Zwietering et al., 1990; Qu et al., 2004; Liu et al., 2005; Mouton and Vinks, 2005; Rao et al., 2018). Several nanomaterials combined with other antibacterial reagents, chemicals, and antibiotics, such as H_2_O_2_, Na_2_CO_3_, acetic acid, methylene blue, toluidine blue, tetracycline, and neomycin, exhibited enhanced synergistic effects against *S. aureus*, *Bacillus subtilis*, *Acinetobacter*, *P. aeruginosa*, and *E. coli* even at concentrations below the MIC (Thakur et al., 2014; Meziani et al., 2016; Dong et al., 2017; Ehsan and Sajjad, 2017; Dong et al., 2018; Abd Elkodous et al., 2020). In the current study, we confirmed the synergistic biocidal effect of C-dots along with tetracycline well below the MIC, showing an enhanced antibacterial effect, reduction in doubling time, and morphological and topological changes on *E. coli* cells. Atomic force microscopy (AFM) studies can provide details about the outer membrane of cells. The earliest surface studies of *E. coli* cells revealed that the force–distance curve was able to detect the surface structure using functionalized probes in AFM imaging and show a detection method of bacteria at the single-cell level (Tanaka et al., 1999; Amro et al., 2000; Miller et al., 2006). After treating with antimicrobial agents such as chitosan, fosfomycin, and other molecules, the images obtained in AFM measurements revealed antibacterial effects, cell wall collapse, and significant morphological changes (Neethirajan and DiCicco, 2014; Eaton et al., 2008; Alves et al., 2010; Ipte and Satpati, 2020; Zhang et al., 2021).

## Materials and Methods

### Materials and Instrumentation

Carbon nanodots obtained from Sigma-Aldrich Inc. were used for this study (Product no. 900414). Antibiotic tetracycline, Luria–Bertani broth, Luria–Bertani agar, Muller–Hinton agar, and Muller–Hinton broth were all research grade (Hi Media laboratories). The instruments used for this study are a spectrophotometer (Chemito UV2300, Shimatdzu Corp.), sonicator (Sonics and materials), shaker, incubator (Classic Scientific), laminar airflow, laboratory centrifuge, scanning electron microscope (ZEISS EVO18), and NTMDT atomic force microscope (NTEGRA). All the glassware used for the experiments were of Borosil, Riviera, Vensil, or Schott Duran.

### Characterization

Carbon dots used for this study were research grade and purchased from Sigma-Aldrich (Product no. 900414). We further characterized the size, absorption spectra, and emission spectra of C-dots using a scanning electron microscope, spectrophotometer, and fluorescence spectrophotometer, respectively (Supplementary Figure S1).

### Biocidal Effect of Tetracycline

The susceptibility of tetracycline on *E. coli* culture was tested on agar plates by a well diffusion assay. In brief, 5.0 ml of 50 μg/ml working stock of tetracycline was prepared and filter-sterilized. From the working stock of tetracycline, different concentrations (0.25 μg/ml, 0.5 μg/ml, 1.0 μg/ml, 1.5 μg/ml, 2.0 μg/ml, 2.5 μg/ml, 3.0 μg/ml, 3.5 μg/ml, 4.0 μg/ml, 4.5 μg/ml, and 5.0 μg/ml) were used for the antibacterial susceptibility assay. An overnight grown culture of *E. coli* (100 µL) spread-plated on Mueller–Hinton agar plates at room temperature (RT), wells were cut in the agar plate with a 5-mm well borer, and 50 µL of each of the tetracycline concentrations, as mentioned before, were added to the individual wells. The aforementioned experiments were performed under sterile conditions in the laminar flow. The plates were incubated at 37°C for 16 h, and the diameter of the zone of inhibition was recorded.

A tetracycline susceptibility test was also carried out in liquid culture to validate the inhibitory effect of the zone of inhibition assay. From the working stock of 50 μg/ml tetracycline, concentrations of tetracycline (0.25 μg/ml, 0.5 μg/ml, 1.0 μg/ml, 1.5 μg/ml, 2.0 μg/ml, 2.5 μg/ml, 3.0 μg/ml 3.5 μg/ml, 4.0 μg/ml, 4.5 μg/ml, 5.0 μg/ml, 5.5 μg/ml, and 6.0 μg/ml) were used by making up the final volume to 5 ml in Muller–Hinton broth. Each test tube was inoculated with 10 µL of the overnight grown culture. The tubes were kept at 37°C on a shaker at 115 RPM for 24 h. The O.D. was recorded at 595 nm.

### Biocidal Effect of C-Dots

From the sonicated stock solution of 2.0 mg/ml of C-dot, 1.0 μg/ml, 2.0 μg/ml, 4.0 μg/ml, 8.0 μg/ml, 16 μg/ml, 32 μg/ml, and 64 μg/ml concentrations were used to assess the antibacterial effect. The 1.0 ml of overnight grown *E. coli* culture was centrifuged, pelleted, washed twice with PBS, and mixed in 1.0 ml PBS. The bacterial culture was treated with the same volume of the C-dot mixture solution to maintain the C-dot concentrations and incubated for 1 h with intermittent mixing at room temperature under visible light conditions. Various studies reported nanomaterial-mediated photodamage of tetracycline. Therefore, we used only visible light in C-dot treatment experiments to avoid nanomaterial-dependent photoinduced tetracycline damage. After incubation, 10 µL of the treated samples were inoculated in individual test tubes containing 5.0 ml of sterile LB. All tubes were incubated in a 37°C incubator shaker at 115 RPM for 8 h. The O.D. was recorded at 595 nm for all the samples.

Four C-dot samples of 8.0 μg/ml, 16 μg/ml, 32 μg/ml, and 64 μg/ml from the treated samples were used to study effect on the bacterial colony count. We serially diluted the treated culture and spread plated the 100 µL of each sample onto the LB agar plates. The plates were incubated at 37°C overnight, and the CFU was calculated from the obtained colony count.

### Synergistic Biocidal Effect of C-Dot and Tetracycline

For the synergistic treatment, 0.1 μg/ml, 0.25 μg/ml, 0.5 μg/ml, 0.75 μg/ml, 1.0 μg/ml, and 1.5 μg/ml concentrations of tetracycline and 2.0 μg/ml, 4.0 μg/ml, 8.0 μg/ml, and 16 μg/ml of C-dots were used. Synergistic doses were maintained by mixing a 1:1 ratio of 500 µL tetracycline +500 μL C-dot. The 1.0 ml of overnight grown *E. coli* culture was centrifuged, pelleted, washed twice with PBS, and mixed in 1.0 ml PBS. The bacterial culture was treated with an equal volume of the tetracycline + C-dot mixture solutions prepared before. And 50 µL of *E. coli* cells were added to each tube to maintain a final volume of 1.0 ml. The culture tubes were incubated for 1 h with intermittent mixing at room temperature. After incubation, 10 µL of the treated samples were inoculated in individual test tubes containing 5.0 ml of sterile LB broth along with the untreated control. All tubes were incubated in a 37°C incubator shaker at 115 RPM for 8 h. The O.D. was recorded at 595 nm for all the samples.

For synergistic treatment, we used 8.0 μg/ml and 16 μg/mL C-dot concentrations with 1.5 μg/ml tetracycline. Two samples were mixed in a 1:1 ratio (500 µL tetracycline +500 μL C-dot) to maintain the aforementioned concentrations and further mixed in 1.0 ml volume of the overnight grown *E. coli* culture. Synergistic treatment was carried out for 1 h at room temperature with intermittent mixing. The treated samples were centrifuged, pelleted, washed twice with PBS, and mixed in 1.0 ml PBS. After treatment, the culture was serially diluted and 100 µL of the treated sample spread onto LB agar plates. The plates were incubated at 37°C overnight; the number of colonies was recorded to calculate CFU.

### Growth Curve Study of Synergistic C-Dot and Tetracycline Treatment

The growth curve of *E. coli* was studied by culturing the bacteria in 25 ml of LB broth overnight at 37°C on a shaker at 115 RPM. Tetracycline and C-dot mixture were prepared, as discussed earlier. A control of LB media together with 1.5 μg/ml tetracycline, 16 μg/mL C-dot, and 1.5 μg/ml tetracycline +16 μg/mL C-dot was used for the growth curve measurement. *E. coli* cells were treated with all the aforementioned dilutions for 1.5 h at 37°C with intermittent mixing. After incubation, 200 µL of each sample was inoculated in conical flasks containing 25 ml of sterile LB broth and kept in an incubator shaker at 37°C at 115 RPM. The O.D. was recorded at 595 nm after every 1-h interval.

### Analysis of Growth Kinetics

Growth rate kinetic analysis of bacteria is oriented toward understanding the cellular process that links the two or more cellular functions, for example, the relationships between growth and cell doubling time, fitted using empirical functions by mathematical modeling based on multiple steps involved in the progression of cell growth. The experimental data sets require the analysis of depth parameter statistics to estimate the precision of the results by applying descriptive methods. Virtually, all of the models developed for kinetic studies are non-linear (Qu et al., 2004). These functional models describe the number of organisms or logarithm of the number of organisms as a function of time. Growth curves obtained in spectroscopic experiments can be modeled using various growth functions such as logistic [Eq. (1)], Gompertz [Eq. (2)], Richards, and Stannard [Eq. (3)]; those are mathematically equivalent (Zwietering et al., 1990; Sprouffske and Wagner, 2016). We process the experimental growth curve data using the following mathematical model described in Eq. 1, Eq. 2, and Eq. 3.

$$y\left(t\right)=\frac{A}{1+\exp\left(\frac{4\mu_{m}}{A\left(\lambda -t\right)+2}\right)},$$ (1)

Gompertz function:

$$y\left(t\right)=Aexp\left(-exp\left(\frac{\mu_{m}exp\left(1\right)}{A}\left(\lambda -t\right)+1\right)\right),$$ (2)

Richard function:

$$y\left(t\right)=A{\left[1+\nu exp\left(1+\nu \right)exp\left(\frac{\mu_{m}}{A}\left(1+\nu \right)\left(1+\frac{1}{\nu }\right)\left(\lambda -t\right)\right)\right]}^{\frac{-1}{\nu }}.$$ (3)

The generation time (*t*_*gen*_) or the population doubling time for logistic growth is related to the growth rate as $t_{gen}=ln\left(2\right)/\mu_{m}$ . The experimental optical density (O.D.) data are a function of time for control without any treatment. Bacterial growth often shows a phase in which the specific growth rate starts at a value of zero and then reaches a maximal value (μm) in a certain period, resulting in a lag time (λ). The growth progress also contains a final phase in which the rate decreases and finally reaches zero, so that an asymptote (A) reached or maximum O.D. We fitted the growth curve of *E. coli*, treated with C-dot, tetracycline, and C-dot + tetracycline, employing the logistic, Gompertz, Richards, and Stannard function using the Levenberg–Marquardt algorithm as implemented in Gnuplot (Williams and Kelley, 2010). These functions are useful to obtain easily interpretable metrics such as population kinetics, the growth rate, the initial population size, and doubling time to summarize microbial growth curve data. In the previous modeled-fit functions, the growth curve is considered a function of O.D. plotted against time. The changes in the growth rate shows sigmoidal behavior (Figure 3), with a lag phase immediately after t = 0, followed by an exponential phase and then a stationary phase.

### Nanoparticles Interaction and Cellular Topology Study

Direct visualization of the cell under high magnification is the best way to assess nanomaterial-dependent toxicity effects in bacterial cells. We used scanning electron microscopy (SEM) and atomic force microscopy (AFM) imaging to study the interaction between the bacteria and nanoparticles, morphological changes, and the topological changes on the cell surface in the independent and synergistically treated samples with C-dots and tetracycline.

### Morphological Study With Scanning Electron Microscopy

The overnight grown *E. coli* cells (2.0 ml) for control, treated culture with C-dot and tetracycline, and their combinations were prepared. *E. coli* cells were grown in LB media for 6 h and centrifuged at 5,000 rpm for 5 min. The supernatant was discarded; the pellet was washed twice with PBS and centrifuged for 5 min at 5,000 rpm. The supernatant was again discarded, and the pellet was resuspended in 50 µL of PBS buffer. Under sterile conditions, 5.0 µL of the *E. coli* sample was spread on a glass slide and allowed to air dry at 20°C. The cells on slides were fixed with 2.5% paraformaldehyde for 20 min. The fixed cells were gently washed twice with PBS and then finally with double distilled water. The fixed cells were post-fixed with 1% osmium tetraoxide for 1 h. The samples were dehydrated by flooding the slide using a series of ethanol concentrations of 35, 50, 75,, and 95% ethanol for 10 min each and finally with absolute alcohol for 10 min. Then the samples were air-dried and dehumidified for 20 min under vacuum and sputter-coated with gold for SEM analyses using a ZEISS EVO 18 SEM imaging system.

### Surface Topology Study by Atomic Force Microscopy

For AFM imaging, 2.0 ml of the overnight grown *E. coli* culture was treated with C-dot and C-dot + tetracycline along with untreated control. Treated cells were centrifuged at 5,000 rpm for 5 min. The supernatant was discarded; the pellet was washed twice with PBS and centrifuged at 5,000 rpm. The collected cell pellets were resuspended in 50 µL PBS, and 20 μL cells spread on the 1.0-cm × 1.0-cm mica sheet. Cells were air-dried and fixed on a mica sheet using 2.5% glutaraldehyde for 30 min. The fixed cell samples were air-dried at 20°C room temperature for AFM analysis. The experiment was carried out with an NTEGRA Prima AFM mounted on an Olympus inverted optical microscope and operated with the NTMDT controller, placed on a *k*-technology vibration isolator. This technique detects the van der Waals forces existing between a probe tip and molecular material (or the surface sample or biological material), scanned along the cantilever tip with a piezo scanner (maximum XYZ scan range of 100 μm × 100 μm x 20 μm). The cantilever tip is attached at one end to a cantilever which is built in at the other end. This technique can generate information detected on surface topography and mechanical properties at the nanometer scale. All data presented in this article were generated with NSG01 series cantilevers (single crystal silicon, N-type, resistivity 0.01–0.025 Ω-cm, antimony-doped), whose spring constant was about 40 N/, with a maximum resonant frequency of 150 kHz and at a scan rate of 0.2–1 Hz. The sample was mounted on the freshly cleaved mica substrate surface 5 mm × 5 mm using the drop-cast method. We performed experiments at 18°C in a dehumidified and acoustic room. We utilized two non-contact atomic force microscopy (NC-AFM) modes; amplitude modulation, which gave height profile, and frequency modulation that provide phase images of the sample. We processed the data obtained in AFM measurements using open source software Gwyddion (Nečas and Klapetek, 2012; Klapetek et al., 2017). The height profile data are processed by subtracting plain surface data to eliminate tilt in the images and perform facet leveling.

## Results

### Biocidal Effect of Tetracycline and C-Dot

**FIGURE 1:**
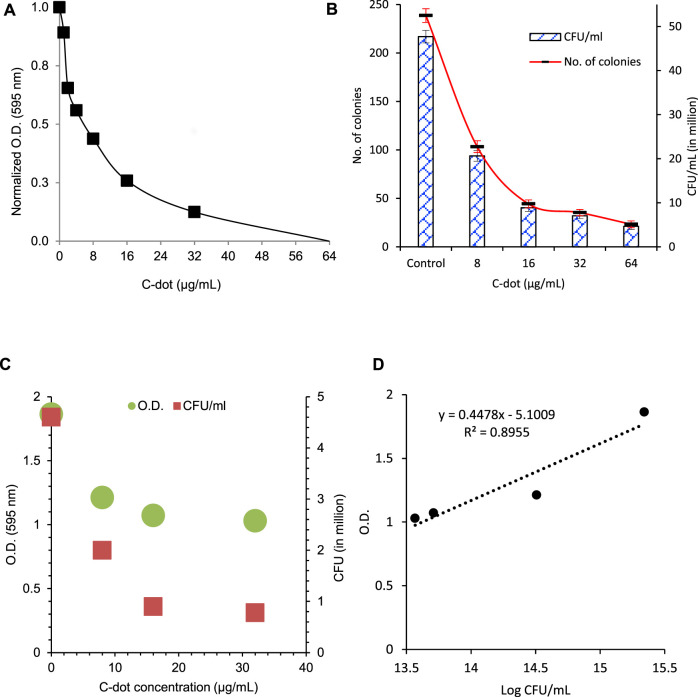
Antibacterial effect of C-dot on *E. coli*. **(A)** Concentration-dependent antibacterial effect in liquid broth. **(B)** Colony formation unit (CFU) count in the agar plate. Supplementary Figure S2 includes all the LB agar plates treated with various concentrations of C-dot. **(C)** CFU and O.D. data of the *E. coli* treated with same concentrations plotted on two Y-axes. **(D)** Correlation between CFU and O.D. of *E. coli* as shown in **(A)** and **(B)**. The correlation value 0.8955 indicates correlation between two data sets.

Tetracycline is a very well-known broad-spectrum antibiotic for the management of bacterial infections. However, the overuse of antibiotics is creating antibiotic resistance against pathogenic strains. Reducing the effective dose of antibiotics is a significant challenge. We have explored the antibacterial efficacy of C-dot. The inhibitory effect was confirmed on *E. coli* cells treated with varying tetracycline concentrations. The results showed inhibition of the *E. coli* growth with tetracycline concentrations of 1.5 μg/ml to 6.0 μg/ml (Supplementary Figure S2 and Supplementary Table S1). Complete inhibition was observed below 1.5 μg/ml concentrations without an inhibition zone. When tested in liquid culture, a decrease in growth was observed up to 1.5 μg/ml concentration. The minimum inhibitory concentration (MIC) for tetracycline against *E. coli* was 1.5 μg/ml. The antibacterial effect on *E. coli* was further studied with a liquid culture using C-dot. The increase in C-dot concentration from 1.0 μg/ml to 32 μg/ml showed growth inhibition and 50% reduction in growth observed at 16 μg/mL C-dot concentration (Figure 1A). The C-dot–treated cells showed a decrease in the number of colonies compared to the control plate, where more than 50% of the colonies decreased at 8.0 μg/mL C-dot concentration (Figure 1B, Supplementary Figure S3 and Supplementary Table S2). CFU decreased as the C-dot concentration increased from 8 μg/ml to 64 μg/mL. A ten time CFU decrease was observed at 64 μg/mL C-dot concentration. A correlation between the O.D. in broth and the CFU of *E. coli* was observed with a correlation coefficient of 0.8955, which indicates good correlation between two independent treatments between O.D. in broth and CFU (Figures 1C,D).

### Synergistic Biocidal Effect of C-Dot and Tetracycline

**FIGURE 2:**
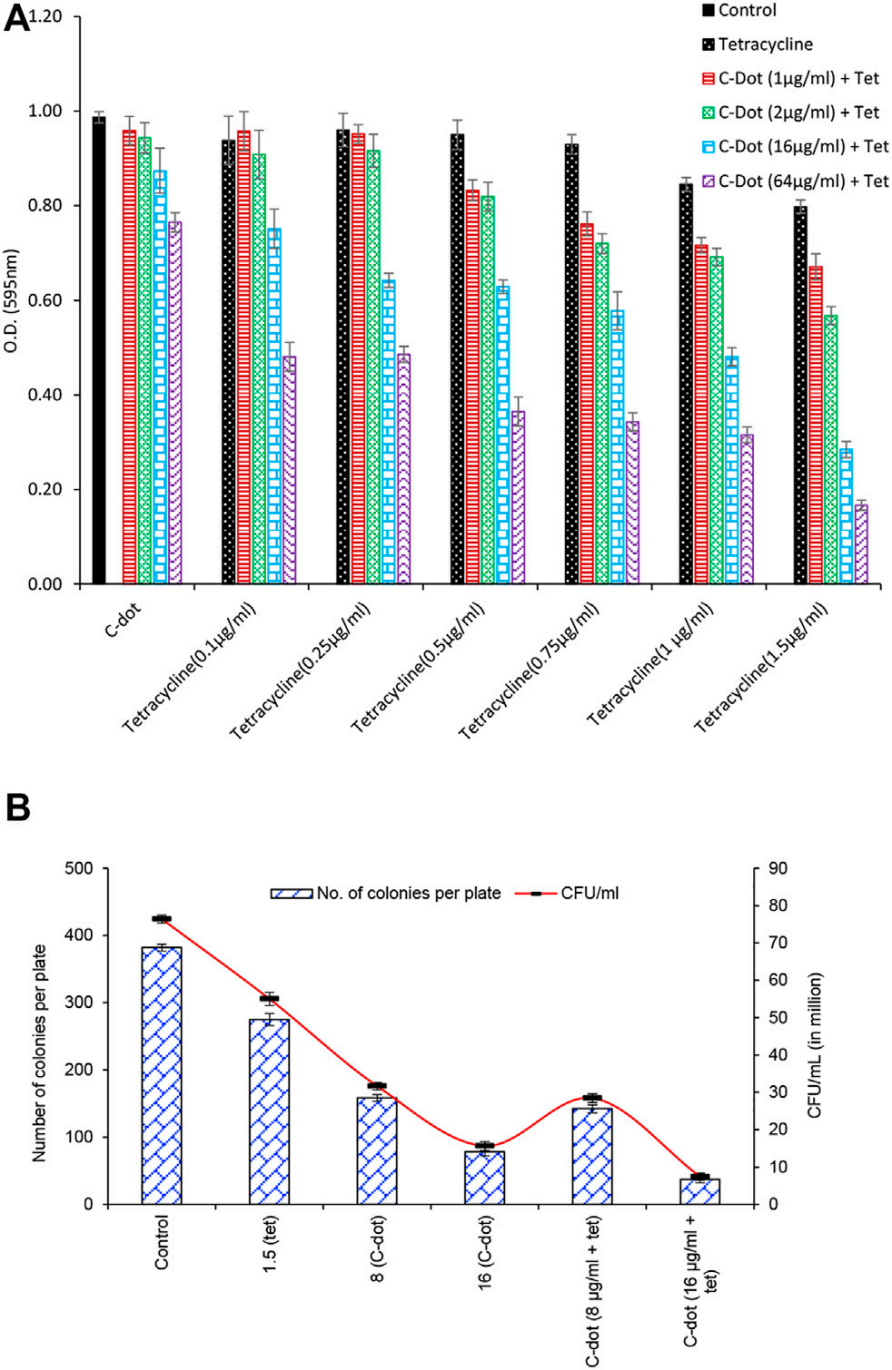
**(A)** Synergistic antibacterial effect of C-dot and tetracycline against *E. coli*. **(B)** CFU count of synergistic antibacterial effect against *E. coli* with tetracycline, C-dot, and in combination. Number of colonies was counted on the LB agar plate and CFU calculated from the count.

Our data for synergistic treatment showed a similar trend in the growth of *E. coli* cells treated with tetracycline and C-dot for the aforementioned concentrations. Significant growth inhibition was observed below and near the MIC of tetracycline together with the MIC of C-dot. The results showed considerable inhibition of the growth of *E. coli* cells with six time lower tetracycline (0.25 μg/ml) along with 0.75 μg/mL C-dot concentration than the MIC (Figure 2A, Supplementary Table S3). Growth of *E. coli* decreased twofold in the 1.5 μg/ml tetracycline (MIC) + 8.0 μg/mL C-dot treated samples compared to independent treatments. The number of colonies reduced eightfold when the concentration of C-dot increased to 16 μg/ml compared to the MIC concentration of tetracycline (Figure 2B, Supplementary Figure S4 and Supplementary Table S3). *E. coli* cell counts were reduced by 30% with 0.75 μg/ml tetracycline along with 1.0 μg/mL C-dot; 50% with 1.5 μg/ml tetracycline with 2.0 μg/mL C-dot; and 75% with 1.5 μg/ml tetracycline with 16 μg/mL C-dot concentrations. In all the aforementioned cases, the concentration of tetracycline was lower or near to the MIC (Figure 2A). These synergistic treatment results indicate a dramatic improvement of the antibacterial efficiency compared to the independent treatment of either tetracycline or C-dot.

### Growth Curve Study for Synergistic Treatment

**FIGURE 3:**
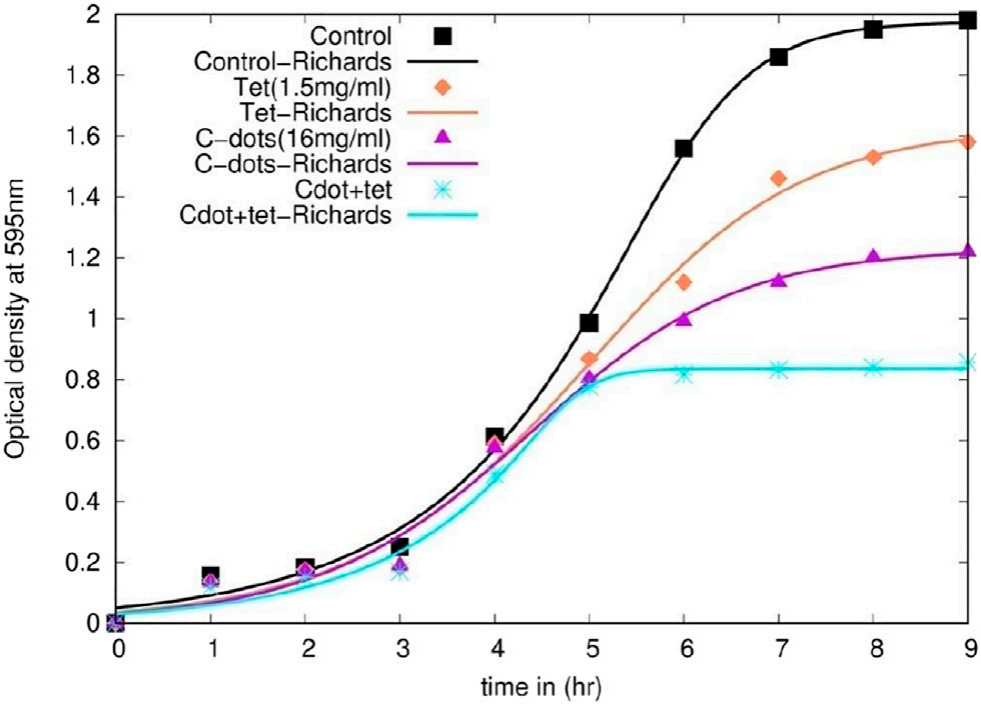
*E. coli* growth curve as obtained by treating the cells with nanoparticles and their combination with tetracycline. The plots with dots indicate experimental optical density for different samples, while with lines indicate non-linear fit to Richard function.

Time-dependent growth retardation and early stationary phase were observed from the growth curve data of synergistic treatment compared to independent treatments either with 1.5 μg/ml tetracycline or 16 μg/mL C-dot (Figure 3 and Supplementary Figure S5A). At 7 h, comparing the control, a 20% decrease in growth of the cells treated with 1.5 μg/ml tetracycline and a 40% reduction in the cells treated with 16 μg/mL C-dot were observed. However, nearly 80% growth reduction in the logarithmic phase was observed on treating the cells with a combination of 16 μg/mL C-dot and 1.5 μg/ml tetracycline. The stationary phase was not attained in both independent treatments at 7 h (Figure 3). These data support the enhanced antibacterial effect in the synergistic treatment. The control culture exhibited a sigmoidal growth curve, and the treated culture sample showed a reduction in growth with an increase in the concentration of the C-dot and tetracycline. The model’s parameters predict that the synergistic treatment with C-dot + tetracycline treatment has a higher inhibitory effect on *E. coli* than the independent treatments*.* The experimental data support the model’s prediction (Figure 3 and Supplementary Figure S4). The model-fit data revealed that the growth rate parameter μ_m_ decreased by 32, 44, and, 42% for logistic function, Gompertz, and Richards function compared to the control (Supplementary Figure S5 and Supplementary Table S1). The Richards function gives a better fit to the experimental growth data (Figure 3).

### Nanoparticle Interaction and Bacterial Morphology Study

#### Scanning Electron Microscopy Analysis

**FIGURE 4:**
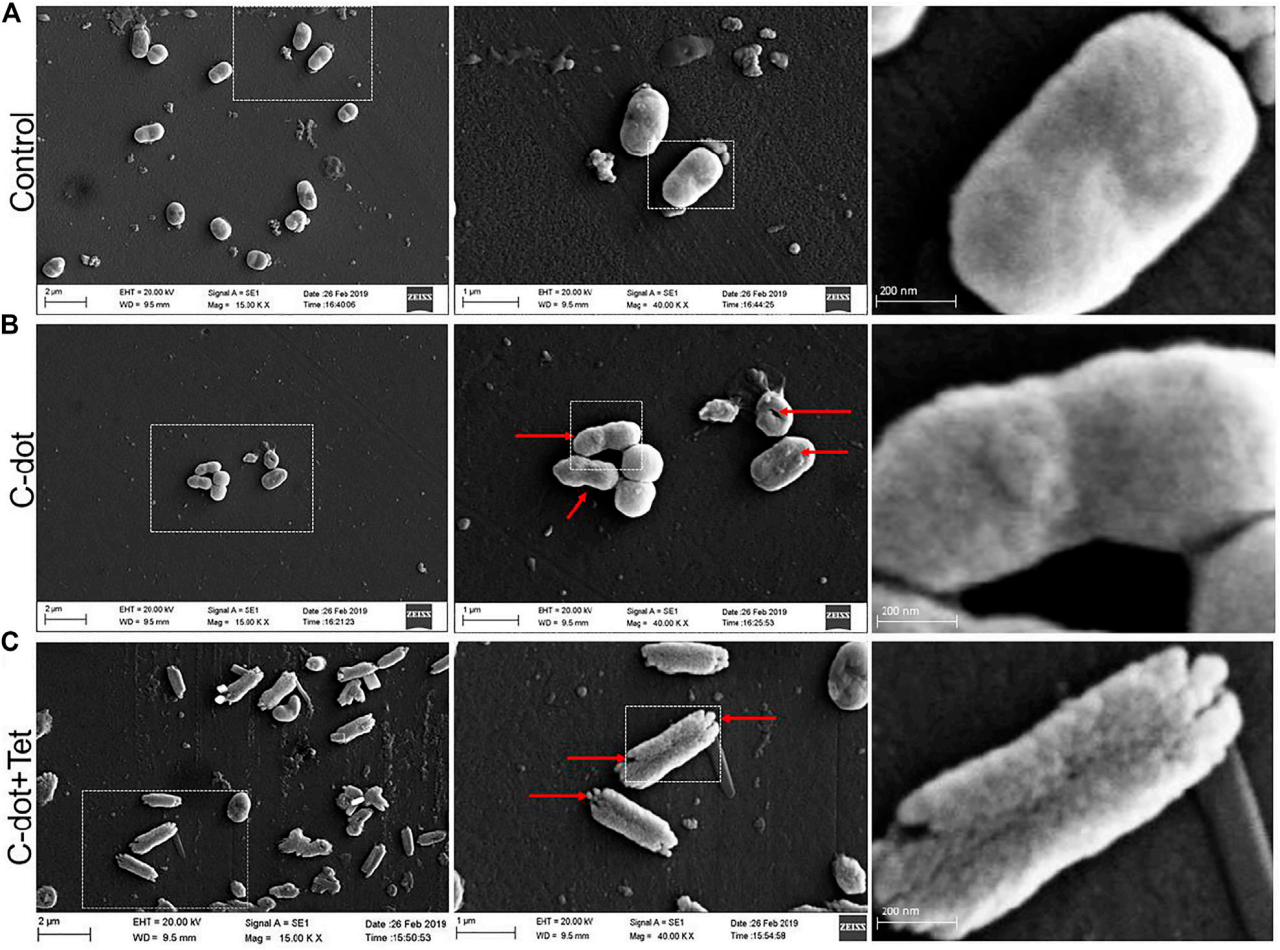
*E. coli* cells observed under scanning electron microscopy (40.00 K X). **(A)** Control *E. coli* cells. **(B)** *E. coli* cells treated with C-dot. **(C)** *E. coli* cells treated with C-dot together with tetracycline. Scale bars in the panels left to right are 2 µm, 1 µm, and 200 nm, respectively.

Morphological changes were observed in C-dot, and C-dot-tetracycline-treated *E. coli* cells. The bacterial cells showed the deposition or binding on the cell surface, deformity in the cellular membranes, and surface roughness (Figure 4). In the samples treated with the combination of C-dot with tetracycline, the *E. coli* cells showed extensive deposition and destruction of cell morphology compared to the cells treated with only C-dot. The red arrow in the image indicates the morphological changes, cell damage, and deposition of C-dot. Compared to the control (Figure 4A), cells treated with C-dot and C-dot–tetracycline clearly show damage and binding on the outer membrane of cells (Figure 4B). However, the synergistic treatment indicates the enhanced biocidal effect, where the cells show severe damage on outer membranes and distorted morphology (Figure 4C).

#### Atomic Force Microscopy Analysis

**FIGURE 5:**
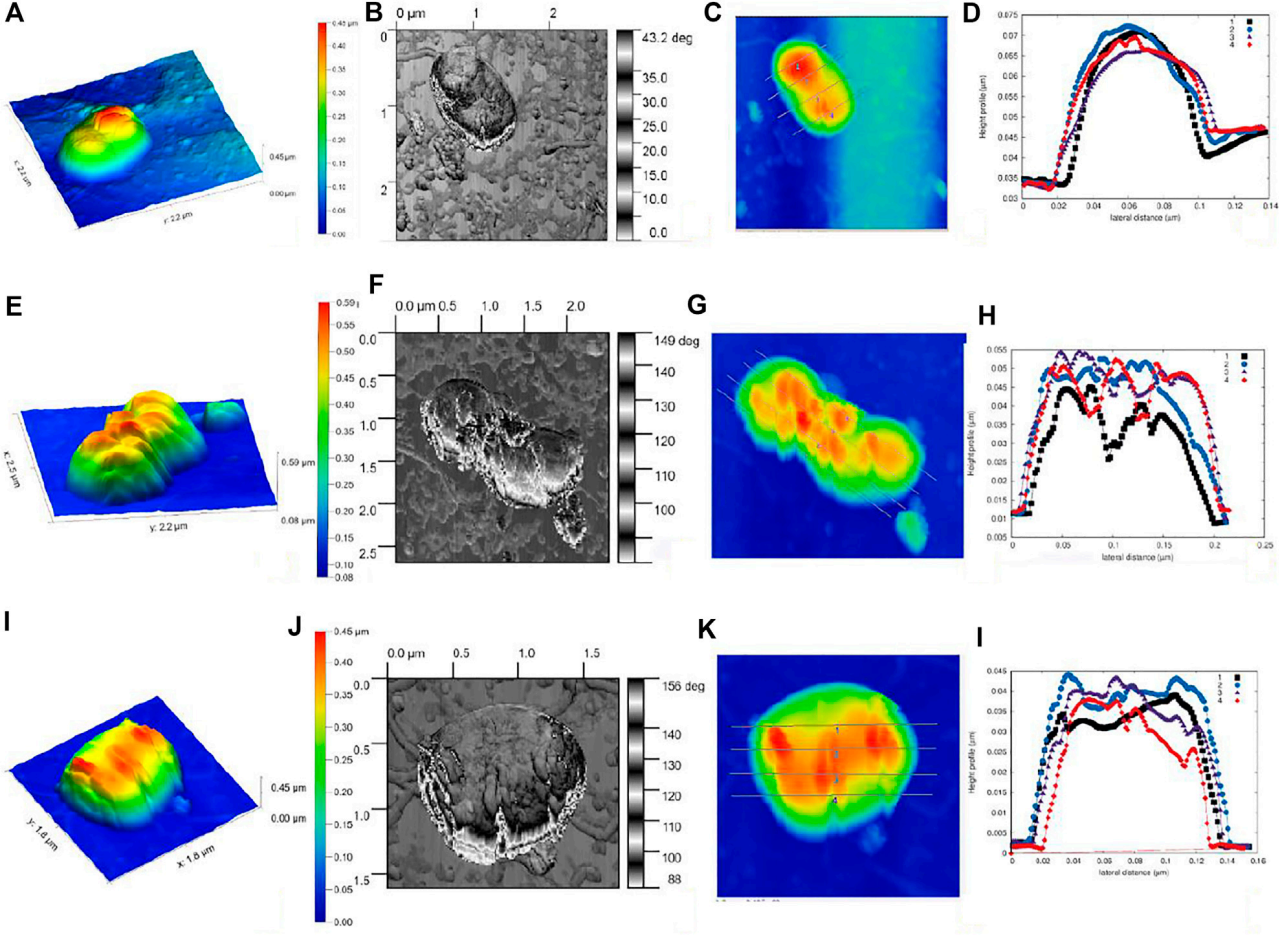
*E. coli* single cell surface topography as mapped under AFM. **(A–D)** Control without any treatment. **(E–H)** *E. coli* cells treated with C-dot. **(I–L)** *E. coli* cells treated with C-dot together with tetracycline, where **(A-E-I)** indicate NC-AFM 3D Height profile, **(B-F-J)** NC-AFM phase contract profile, **(C-G-K)** linear path mapping across single cell, **(D-H-L)** height profile graph of surface roughness for *E. coli* samples.

The AFM studies of the three-dimensional imaging of the *E. coli* bacterial samples treated with C-dot and tetracycline revealed morphological/topological changes. The plotted graph for cell height and surface roughness suggests the high degree of roughness on C-dot–treated cells compared to control cells (Figure 5 and Supplementary Figure S6). We performed AFM measurements under ambient conditions (air) at a non-/contact intermittent mode to get the advantage of the harmonic oscillator response behavior of the AFM cantilever. This operating mode measures minimal force (quantum chemical van der Waals force) that prevents the indentation of bacteria by the AFM tip. This will protect the outer membrane of the *E. coli* cells, which is reflected in the observed phase-contrast and height images. Imaging of *E. coli* under physiological conditions would make more sense, but this will lower the accuracy of the nanoscale resolution on the outer membrane. Phase images (or frequency modulation mode) have been used to achieve the detail information of outer membrane which is not possible in height imaging (or amplitude mode). The Phase imaging mode is the phase difference between the exciting signal and the response of the cantilever. This mode is susceptible to the increase in interaction between the AFM tip and the surface. The dissipative processes happening in the AFM tip allow the study of the electromechanical properties of bacterial membranes at nanoscale. The results obtained in the present experiment are shown in Figure 5. The height information has been extracted from a linear map across a sample cell in the control cell. These data predict that the control cell has a length of ∼1.1 μm with a height of about 38 nm and a width of 80 nm (Figures 5A–D). The (linear path) cross section across the control cell shows the smooth topological distribution, with vertical height distribution (ΔZ) of 8.0 nm. However, the C-dot– and C-dot + tetracycline–treated samples show ΔZ distribution of ∼31 and ∼25 nm, respectively. These data indicate the higher morphological change with C-dot + tetracycline synergistic treatment (Figures 5E–L). The average line profile over multiple cells is shown in Supplementary Figure S6B. A minor deviation is observed for the peak of line profile cross section ∼ 8 nm for control cells, while ∼22 and ∼30 nm for cells treated with C-dot and C-dot + tetracycline.

## Discussion

Due to the emerging antibiotic resistance, there is a need to reduce the exposure and the heavy dose of antibiotics or replace other biocompatible antibacterial materials. The problem of antibiotic resistance has become a global issue, especially in the public health sector. To combat this, nanoparticles and their combination with other broad-spectrum bactericidal agents can be a potential solution. In the present study, we have used a tetracycline and C-dot combination to minimize biocidal treatment. Tetracycline inhibits bacterial protein synthesis and prevents the association of aminoacyl-tRNA with the bacterial ribosome (Chopra et al., 1992). C-dot is a biocompatible nanomaterial with a potent antibacterial effect. This study revealed a synergistic approach using antibiotics and C-dots below their MIC, resulting in enhanced bactericidal properties against *E. coli*. The proposed mechanism for the toxicity of C-dot is due to the effect of photoinduced electrons and holes responsible for their antimicrobial properties under visible light exposure (Lim et al., 2015; Meziani et al., 2016). In case of synergistic antibacterial treatment, bactericidal activity was observed with a ten time lower concentration of the MIC of tetracycline (0.1 μg/ml, 0.25 μg/ml, and 0.5 μg/ml) combined with low doses of C-dots (1.0 μg/ml, 2.0 μg/ml). An enhanced inhibitory effect was observed when the concentration of C-dot increased to 16 μg/ml and 32 μg/ml. The combination of C-dot and tetracycline indicates an effective bactericidal effect.

The stationary phase was achieved in 5 h compared to the independent treatment, where the bacteria were still in their log phase even after 10 h. The growth rate analysis data predict that mathematical models such as logistic, Gompertz, and Richards function models predict the experimental data very well (Table 1). The growth rate parameter (μ_m_), which controls the steepness of the function, is the primary variable that describes the cellular growth. The growth rate parameter (μ_m_) shows a significant decrease (∼40%) in C-dot and tetracycline samples compared to the control. Attachment of C-dots and cell damage observed with C-dot treatment in the SEM study indicated that the particle and cell surface interaction was prominent with C-dot treatment. There were drastic morphological changes and cell damage observed in the *E. coli* cells in the synergistic treatment of C-dot together with tetracycline. The AFM study with C-dot and tetracycline also indicates outer membrane roughness in the height profile (amplitude imaging) and cellular morphological changes in phase images compared to the control. The exact mechanism of how these particles interact with the cell surface either directly or indirectly still remains unexplored. This study needs to be extended further with antibiotic-resistant pathogens to identify the intricate dose and other parameters for synergistic use. Thus, this study establishes a framework to combat antibiotic-resistant bacteria, presently demonstrated using *E. coli* as a model organism and inhibition of growth with a much lower MIC dose of tetracycline concentration when used in combination with C-dot.

**TABLE 1:** Parameters (A, λ, and μ_m_) of non-linear fit for various functions used in the article.

	—	A	μ_m_	λ	ν
Control	Logistic	2.06	0.49	2.84	—
	Gompertz	2.2	0.46	2.65	—
	Richards	1.97	0.67	3.23	3.16
	Stannard	1.97	0.67	3.23	3.16
Tet	Logistic	1.65	0.33	2.46	—
	Gompertz	1.81	0.31	2.22	—
	Richards	1.62	0.38	2.56	1.48
	Stannard	1.62	0.38	2.56	1.48
C-dot	Logistic	1.24	0.27	2.06	—
	Gompertz	1.31	0.25	1.87	—
	Richards	1.22	0.29	2.12	1.33
	Stannard	1.22	0.29	2.12	1.33
C-dot + tet	Logistic	0.85	0.28	2.23	—
	Gompertz	0.86	0.33	2.43	—
	Richards	0.84	0.34	2.36	2.1
	Stannard	0.84	0.34	2.36	2.1

## Conclusion

This study demonstrates that the C-dot with tetracycline is effective for synergistic antibacterial treatment with a ten time lower tetracycline concentration than the MIC. Our SEM and AFM imaging data prove the cellular damage and change in the cell topology. We also ascertain our work with logistic, Gompertz, and Richards mathematical model, which suggest that all the models reveal the antibacterial effect as seen in the bacterial growth curve. The Richards model gives the best fit. These models will help understand and extrapolate the minimal effects on bacterial growth. We summarize that C-dot and tetracycline are ideal candidates for antibacterial cocktails and possibly effective treatment for pathogenic MDR bacterial strains with minimal antibiotic dose.

## Data Availability

The original contributions presented in the study are included in the article/Supplementary Material, further inquiries can be directed to the corresponding authors.

## Abbreviations

AFM, atomic force microscopy; C-dot, carbon nanodot; CFU, colony formation unit; LB, Luria–Bertani; MDR, multidrug resistance; MIC, minimum inhibitory concentration; NP, nanoparticle; OD, optical density;RPM, rotation per minute; SEM, scanning electron microscopy.

## References

[B1] Abd Elkodous M. El-Sayyad G. S. Youssry S. M. Nada H. G. Gobara M. Elsayed M. A. (2020). Carbon-dot-loaded CoxNi1−xFe2O4; X = 0.9/SiO2/TiO2 Nanocomposite with Enhanced Photocatalytic and Antimicrobial Potential: An Engineered Nanocomposite for Wastewater Treatment. Sci. Rep. 10 (1), 1–22. 10.1038/s41598-020-68173-1 32661303 PMC7358215

[B2] Akhavan O. Ghaderi E. Esfandiar A. (2011). Wrapping Bacteria by Graphene Nanosheets for Isolation from Environment, Reactivation by Sonication, and Inactivation by Near-Infrared Irradiation. J. Phys. Chem. B 115, 6279–6288. 10.1021/jp200686k 21513335

[B3] Alves C. S. Melo M. N. Franquelim H. G. Ferre R. Planas M. Feliu L. (2010). *Escherichia coli* Cell Surface Perturbation and Disruption Induced by Antimicrobial Peptides BP100 and pepR. J. Biol. Chem. 285 (36), 27536–27544. 10.1074/jbc.m110.130955 20566635 PMC2934620

[B4] Amro N. A. Kotra L. P. Wadu-Mesthrige K. Bulychev A. Mobashery S. Liu G.-y. (2000). High-Resolution Atomic Force Microscopy Studies of theEscherichiacoliOuter Membrane: Structural Basis for Permeability. Langmuir 16 (6), 2789–2796. 10.1021/la991013x

[B5] Beyth N. Houri-Haddad Y. Domb A. Khan W. Hazan R. (2015). Alternative Antimicrobial Approach: Nano-Antimicrobial Materials. Evidence-Based Complement. Altern. Med. 2015, 1–16. 10.1155/2015/246012 PMC4378595 25861355

[B6] Cao L. Meziani M. J. Sahu S. Sun Y.-P. (2013). Photoluminescence Properties of Graphene versus Other Carbon Nanomaterials. Acc. Chem. Res. 46, 171–180. 10.1021/ar300128j 23092181

[B7] Chopra I. Hawkey P. M. Hinton M. (1992). Tetracyclines, Molecular and Clinical Aspects. J. Antimicrob. Chemother. 29, 245–277. 10.1093/jac/29.3.245 1592696

[B8] Chun S. Muthu M. Gansukh E. Thalappil P. Gopal J. (2016). The Ethanopharmacological Aspect of Carbon Nanodots in Turmeric Smoke. Sci. Rep. 6 (1):35586. 10.1038/srep35586 27805007 PMC5090208

[B9] Daeihamed M. Dadashzadeh S. Haeri A. Akhlaghi M. (2016). Potential of Liposomes for Enhancement of Oral Drug Absorption. Curr. Drug. Deliv. 13 (999), 1. 10.2174/1567201813666160115125756 26768542

[B10] Dong X. Awak M. A. TomLinson N. Tang Y. Sun Y.-P. Yang L. (2017). Antibacterial Effects of Carbon Dots in Combination with Other Antimicrobial Reagents. PLoS ONE 12 (9), e0185324–16. 10.1371/journal.pone.0185324 28934346 PMC5608398

[B11] Dong X. Bond A. E. Pan N. Coleman M. Tang Y. Sun Y.-P. (2018). Synergistic Photoactivated Antimicrobial Effects of Carbon Dots Combined with Dye Photosensitizers. I. J. Nanomed. 13, 8025–8035. 10.2147/IJN.S183086 PMC6267493 30568443

[B12] Eaton P. Fernandes J. C. Pereira E. Pintado M. E. Xavier Malcata F. (2008). Atomic Force Microscopy Study of the Antibacterial Effects of Chitosans on *Escherichia coli* and *Staphylococcus aureus* . Ultramicroscopy 108 (10), 1128–1134. 10.1016/j.ultramic.2008.04.015 18556125

[B13] Ehsan S. Sajjad M. (2017). Bioinspired Synthesis of Zinc Oxide Nanoparticle and its Combined Efficacy with Different Antibiotics against Multidrug Resistant Bacteria. J. Biomat. Nano. Biotech. 08, 159–175. 10.4236/jbnb.2017.82011

[B14] Fernando K. A. S. Sahu S. Liu Y. Lewis W. K. Guliants E. A. Jafariyan A. (2015). Carbon Quantum Dots and Applications in Photocatalytic Energy Conversion. ACS Appl. Mater. Inter. 7, 8363–8376. 10.1021/acsami.5b00448 25845394

[B15] Gholipourmalekabadi M. Mobaraki M. Ghaffari M. Zarebkohan A. Omrani V. F. Urbanska A. M. (2017). Targeted Drug Delivery Based on Gold Nanoparticle Derivatives. Curr. Pharm. Des. 23 (20), 2918–2929. 10.2174/1381612823666170419105413 28425863

[B16] Hu S. Trinchi A. Atkin P. Cole I. (2015). Tunable Photoluminescence across the Entire Visible Spectrum from Carbon Dots Excited by white Light. Angew. Chem. Int. Ed. 54, 2970–2974. 10.1002/anie.201411004 25589468

[B17] Ipte P. R. Satpati A. K. (2020). Probing the Interaction of Ciprofloxacin and *E. coli* by Electrochemistry, Spectroscopy and Atomic Force Microscopy. Biophysical Chem. 266, 106456. 10.1016/j.bpc.2020.106456 32835912

[B18] Jelinek R. (2017). Carbon-dot Synthesis. in Carbon Nanostructures, 5–27. 10.1007/978-3-319-43911-2_2

[B19] Klapetek P. Yacoot A. Grolich P. Valtr M. Nečas D. (2017). Gwyscan: A Library to Support Non-equidistant Scanning Probe Microscope Measurements. Meas. Sci. Technol. 28, 034015. 10.1088/1361-6501/28/3/034015

[B20] LeCroy G. E. Yang S.-T. Yang F. Liu Y. Fernando K. A. S. Bunker C. E. (2016). Functionalized Carbon Nanoparticles: Syntheses and Applications in Optical Bioimaging and Energy Conversion. Coord. Chem. Rev. 320-321, 66–81. 10.1016/j.ccr.2016.02.017

[B21] Li J. Singh V. V. Sattayasamitsathit S. Orozco J. Kaufmann K. Dong R. (2014a). Water-driven Micromotors for Rapid Photocatalytic Degradation of Biological and Chemical Warfare Agents. ACS Nano 8, 11118–11125. 10.1021/nn505029k 25289459

[B22] Li J. Wang G. Zhu H. Zhang M. Zheng X. Di Z. (2014b). Antibacterial Activity of Large-Area Monolayer Graphene Film Manipulated by Charge Transfer. Sci. Rep. 4 (1), 4359. 10.1038/srep04359 24619247 PMC3950808

[B23] Lim S. Y. Shen W. Gao Z. (2015). Carbon Quantum Dots and Their Applications. Chem. Soc. Rev. 44, 362–381. 10.1039/c4cs00269e 25316556

[B24] Liu P. Rand K. H. Obermann B. Derendorf H. (2005). Pharmacokinetic-pharmacodynamic Modelling of Antibacterial Activity of Cefpodoxime and Cefixime in *In Vitro* Kinetic Models. Int. J. Antimicrob. Agents 25, 120–129. 10.1016/j.ijantimicag.2004.09.012 15664481

[B25] Liu X. Yang Y. Li H. Yang Z. Fang Y. (2021). Visible Light Degradation of Tetracycline Using Oxygen-Rich Titanium Dioxide Nanosheets Decorated by Carbon Quantum Dots. Chem. Eng. J. 408 (October), 127259. 10.1016/j.cej.2020.127259

[B26] López-Peñalver J. J. Sánchez-Polo M. Gómez-Pacheco C. V. Rivera-Utrilla J. (2010). Photodegradation of Tetracyclines in Aqueous Solution by Using UV and UV/H2O2 Oxidation Processes. J. Chem. Technol. Biotechnol. 85 (10), 1325–1333. 10.1002/jctb.2435

[B27] Luo P. G. Yang F. Yang S.-T. Sonkar S. K. Yang L. Broglie J. J. (2014). Carbon-based Quantum Dots for Fluorescence Imaging of Cells and Tissues. RSC Adv. 4, 10791. 10.1039/c3ra47683a

[B28] Maas M. (2016). Carbon Nanomaterials as Antibacterial Colloids. Materials 9, 617. 10.3390/ma9080617 PMC5509023 28773737

[B29] Meziani M. J. Dong X. Zhu L. Jones L. P. Lecroy G. E. Yang F. (2016). Visible-Light-Activated Bactericidal Functions of Carbon "Quantum" Dots. ACS Appl. Mater. Inter. 8, 10761–10766. 10.1021/acsami.6b01765 PMC5017886 27064729

[B30] Miller E. Garcia T. Hultgren S. Oberhauser A. F. (2006). The Mechanical Properties of *E. coli* Type 1 Pili Measured by Atomic Force Microscopy Techniques. Biophysical J. 91 (10), 3848–3856. 10.1529/biophysj.106.088989 PMC1630485 16950852

[B31] Mouton J. W. Vinks A. A. (2005). Pharmacokinetic/Pharmacodynamic Modelling of Antibacterials *In Vitro* and *In Vivo* Using Bacterial Growth and Kill Kinetics. Clin. Pharmacokinet. 44, 201–210. 10.2165/00003088-200544020-00005 15656698

[B32] Mühling M. Bradford A. Readman J. W. Somerfield P. J. Handy R. D. (2009). An Investigation into the Effects of Silver Nanoparticles on Antibiotic Resistance of Naturally Occurring Bacteria in an Estuarine Sediment. Mar. Environ. Res. 68, 278–283. 10.1016/j.marenvres.2009.07.001 19665221

[B33] Muktha H. Sharath R. Kottam N. Smrithi S. P. Samrat K. Ankitha P. (2020). Green Synthesis of Carbon Dots and Evaluation of its Pharmacological Activities. BioNanoSci. 10 (3), 731–744. 10.1007/s12668-020-00741-1

[B34] Nagamine M. Osial M. Jackowska K. Krysinski P. Widera-Kalinowska J. (2020). Tetracycline Photocatalytic Degradation under CdS Treatment. Jmse 8 (7), 483. 10.3390/jmse8070483

[B35] Najjar M. B. Kashtanov D. Chikindas M. L. (2007). ?-Poly-l-lysine and Nisin A Act Synergistically against Gram-Positive Food-Borne Pathogens Bacillus Cereus and Listeria Monocytogenes. Lett. Appl. Microbiol. 45, 13–18. 10.1111/j.1472-765X.2007.02157.x 17594454

[B36] Naseri N. Valizadeh H. Zakeri-Milani P. (2015). Solid Lipid Nanoparticles and Nanostructured Lipid Carriers: Structure, Preparation and Application. Adv. Pharm. Bull. 5, 305–313. 10.15171/apb.2015.043 26504751 PMC4616893

[B37] Nečas D. Klapetek P. (2012). Gwyddion: An Open-Source Software for SPM Data Analysis. Cent. Eur. J. Phys. 10, 181–188. 10.2478/s11534-011-0096-2

[B38] Neethirajan S. DiCicco M. (2014). Atomic Force Microscopy Study of the Antibacterial Effect of Fosfomycin on Methicillin-Resistant Staphylococcus Pseudintermedius. Appl. Nanosci 4 (6), 703–709. 10.1007/s13204-013-0256-3

[B39] Pelgrift R. Y. Friedman A. J. (2013). Nanotechnology as a Therapeutic Tool to Combat Microbial Resistance. Adv. Drug Deliv. Rev. 65, 1803–1815. 10.1016/j.addr.2013.07.011 23892192

[B40] Pissuwan D. Niidome T. Cortie M. B. (2011). The Forthcoming Applications of Gold Nanoparticles in Drug and Gene Delivery Systems. J. Controlled Release 149, 65–71. 10.1016/j.jconrel.2009.12.006 20004222

[B41] Qu Z. Weiss J. N. MacLellan W. R. (2004). Coordination of Cell Growth and Cell Division: A Mathematical Modeling Study. J. Cel Sci. 117, 4199–4207. 10.1242/jcs.01294 15280433

[B42] Rao G. G. Li J. Garonzik S. M. Nation R. L. Forrest A. (2018). Assessment and Modelling of Antibacterial Combination Regimens. Clin. Microbiol. Infect. 24, 689–696. 10.1016/j.cmi.2017.12.004 29269090

[B43] Righetto M. Privitera A. Fortunati I. Mosconi D. Zerbetto M. Curri M. L. (2017). Spectroscopic Insights into Carbon Dot Systems. J. Phys. Chem. Lett. 8, 2236–2242. 10.1021/acs.jpclett.7b00794 28471190

[B44] Rocha C. G. Dargam T. G. Latgé A. (2002). Carbon Nanotube Quantum Dots. Physica Status Solidi (B) Basic Res. 232 (1), 37–43. 10.1002/1521-3951(200207)232:1<37::aid-pssb37>3.0.co;2-p

[B45] Semeraro P. Bettini S. Sawalha S. Pal S. Licciulli A. Marzo F. (2020). Photocatalytic Degradation of Tetracycline by ZnO/γ-Fe2O3 Paramagnetic Nanocomposite Material. Nanomaterials 10 (8), 1458–1512. 10.3390/nano10081458 PMC7466472 32722422

[B46] Sprouffske K. Wagner A. (2016). Growthcurver: An R Package for Obtaining Interpretable Metrics from Microbial Growth Curves. BMC Bioinformatics 17. 10.1186/s12859-016-1016-7 PMC4837600 27094401

[B47] Sun Y.-P. Zhou B. Lin Y. Wang W. Fernando K. A. S. Pathak P. (2006). Quantum-sized Carbon Dots for Bright and Colorful Photoluminescence. J. Am. Chem. Soc. 128, 7756–7757. 10.1021/ja062677d 16771487

[B48] Tanaka T. Nakamura N. Matsunaga T. (1999). Atomic Force Microscope Imaging of *Escherichia coli* Cell Using Anti-*E. coli* Antibody-Conjugated Probe (In Aqueous) Solutions. Electrochimica acta 44 (21-22), 3827–3832. 10.1016/s0013-4686(99)00089-4

[B49] Thakur M. Pandey S. Mewada A. Patil V. Khade M. Goshi E. (2014). Antibiotic Conjugated Fluorescent Carbon Dots as a Theranostic Agent for Controlled Drug Release, Bioimaging, and Enhanced Antimicrobial Activity. J. Drug Deliv. 2014, 1–9. 10.1155/2014/282193 PMC3976943 24744921

[B50] Thukral D. Dumoga S. Mishra A. (2014). Solid Lipid Nanoparticles: Promising Therapeutic Nanocarriers for Drug Delivery. Cdd 11, 771–791. 10.2174/156720181106141202122335 25469779

[B51] Williams T. Kelley C. (2010). Gnuplot 4.4: an Interactive Plotting Program. Available at: Http//Sourceforge. Net/Projects/Gnuplot (Accessed August, 2020).

[B52] Xin Q. Shah H. Nawaz A. Xie W. Akram M. Z. Batool A. (2019). Antibacterial Carbon‐Based Nanomaterials. Adv. Mater. 31, 1804838. 10.1002/adma.201804838 30379355

[B53] Xu W. Lai S. Pillai S. C. Chu W. Hu Y. Jiang X. (2020). Visible Light Photocatalytic Degradation of Tetracycline with Porous Ag/graphite Carbon Nitride Plasmonic Composite: Degradation Pathways and Mechanism. J. Colloid Interf. Sci. 574, 110–121. 10.1016/j.jcis.2020.04.038 32311534

[B54] Yang J. Zhang X. Ma Y.-H. Gao G. Chen X. Jia H.-R. (2016). Carbon Dot-Based Platform for Simultaneous Bacterial Distinguishment and Antibacterial Applications. ACS Appl. Mater. Inter. 8, 32170–32181. 10.1021/acsami.6b10398 27786440

[B55] Zhang X. Yang C. Xi T. Zhao J. Yang K. (2021). Surface Roughness of Cu-Bearing Stainless Steel Affects its Contact-Killing Efficiency by Mediating the Interfacial Interaction with Bacteria. ACS Appl. Mater. Inter. 13 (2), 2303–2315. 10.1021/acsami.0c19655 33395246

[B56] Zou X. Zhang L. Wang Z. Luo Y. (2016). Mechanisms of the Antimicrobial Activities of Graphene Materials. J. Am. Chem. Soc. 138, 2064–2077. 10.1021/jacs.5b11411 26824139

[B57] Zwietering M. H. Jongenburger I. Rombouts F. M. van 't Riet K. (1990). Modeling of the Bacterial Growth Curve. Appl. Environ. Microbiol. 56, 1875–1881. 10.1128/aem.56.6.1875-1881.1990 16348228 PMC184525

