# Supplementary material for: Synergistic Antibacterial Potential and Cell Surface Topology Study of Carbon Nanodots and Tetracycline Against E. coli
Source: Front Bioeng Biotechnol. 2021 Oct 5;9:626276. doi: 10.3389/fbioe.2021.626276 (PMC8524088; doi:10.3389/fbioe.2021.626276)
Supplement: Supplementary file 1 [file Presentation1.pdf]

SUPPLEMENTARY DATA

Synergistic antibacterial potential and cell surface topology study of carbon nanodots and tetracycline against *E. coli*

Dhermendra K. Tiwari[1,*], Gargi Jha[1], Manisha Tiwari[1], and Savita Kerkar[1], Suman Das[1], and Vivekanand V. Gobre[2,*]

[1]Department of Biotechnology, Faculty of Life Sciences and Environment, Goa University, Taleigao Plateau, Goa, India 403206

[2]School of Chemical Sciences, Goa University. Taleigao Plateau, Goa, India 403206

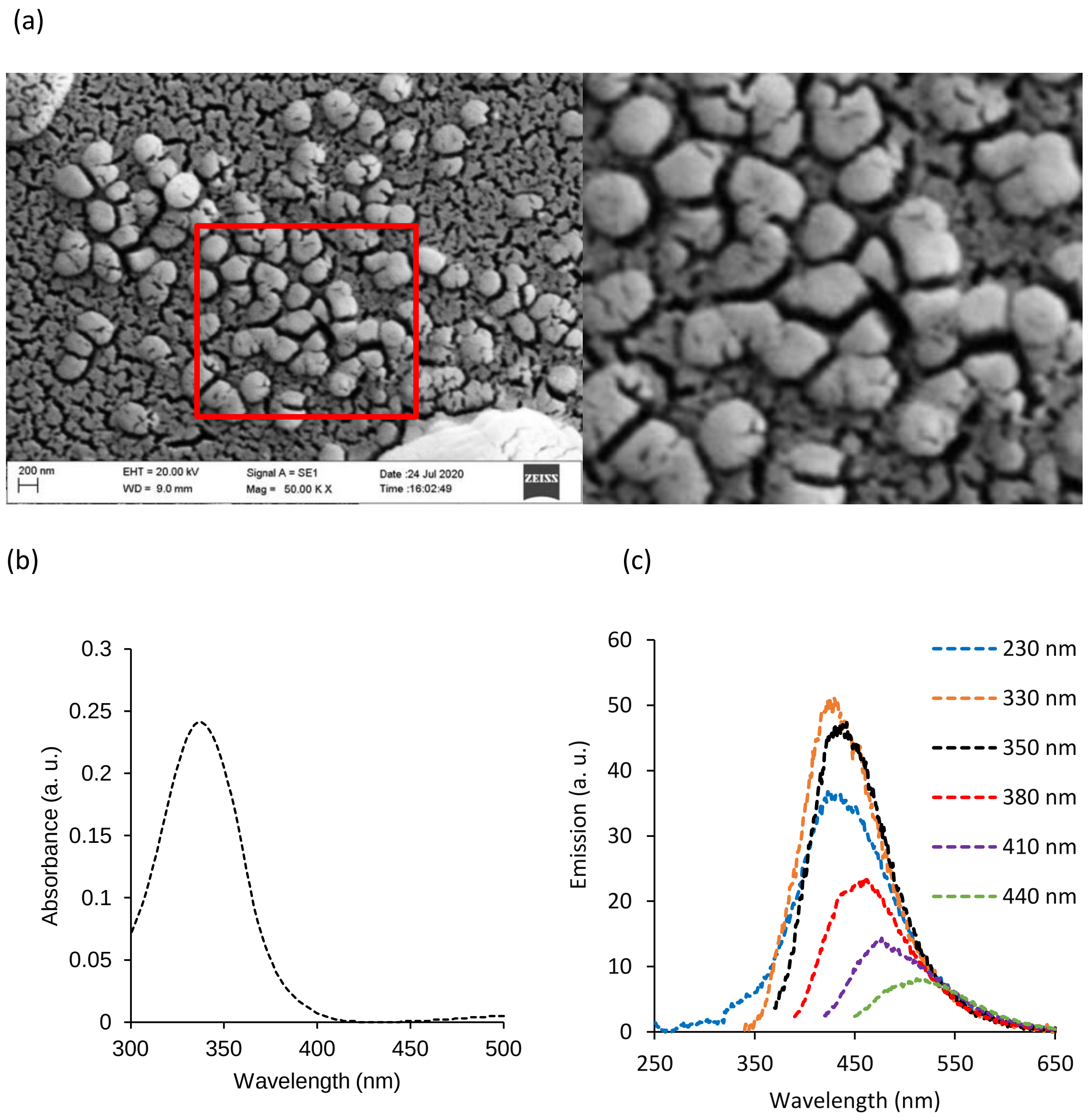

Supple. Figure 1 | Characterization of C-dot. (a) Scanning electron microscopy image, (b) absorption spectra and (c) emission spectra at different excitation wavelengths.

(a)

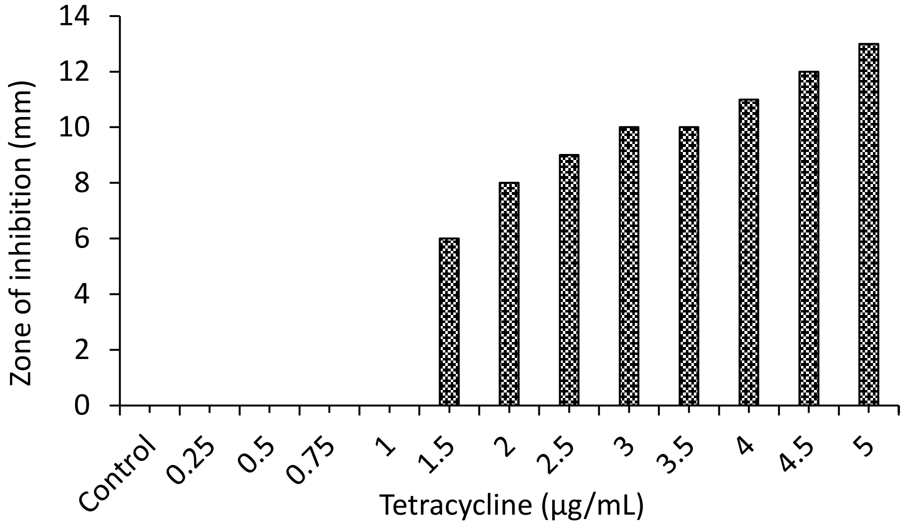

(b)

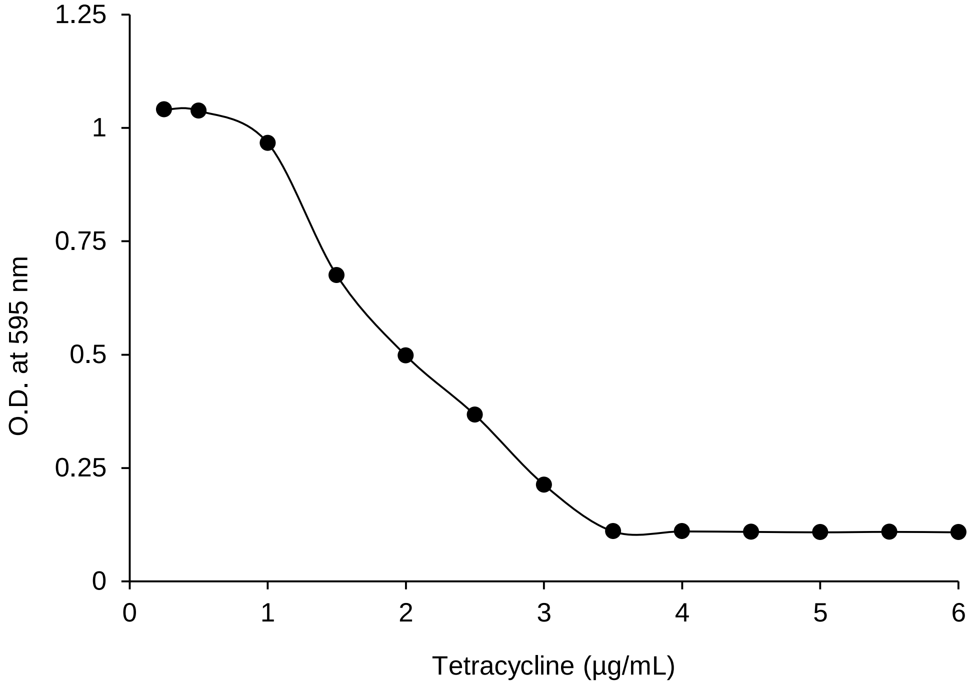

Suppl. Figure 2 | Zone of inhibition and liquid broth assay of various tetracycline concentrations against *E. coli* culture.

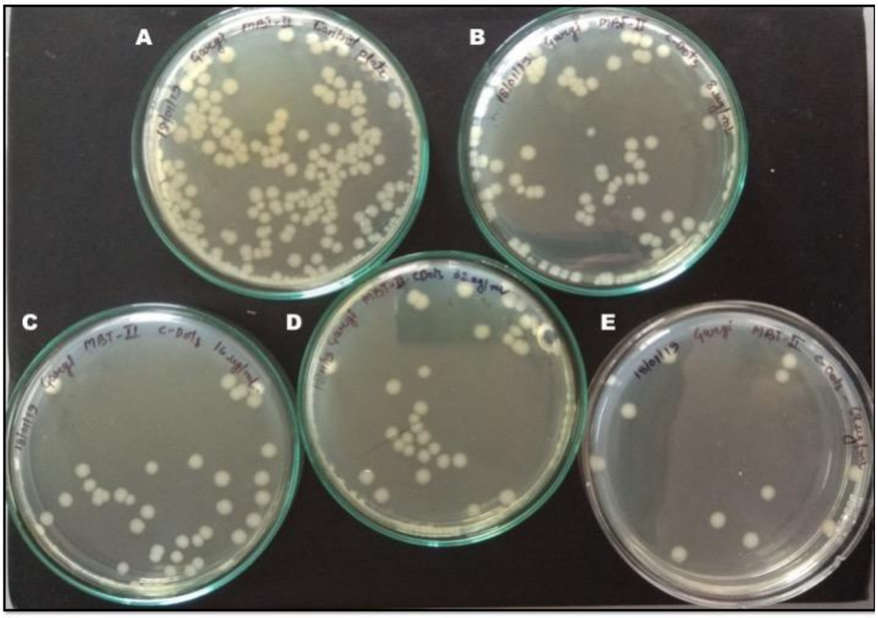

Suppl. Figure 3 | LB agar plates for CFU assay. Cells were treated with various concentrations of C-dot. A) Control plate, B) 8 µg/mL C-dot, C) 16 µg/mL C-dot, D) 32 µg/mL C-dot treated cells, E) 64 µg/mL C-dot.

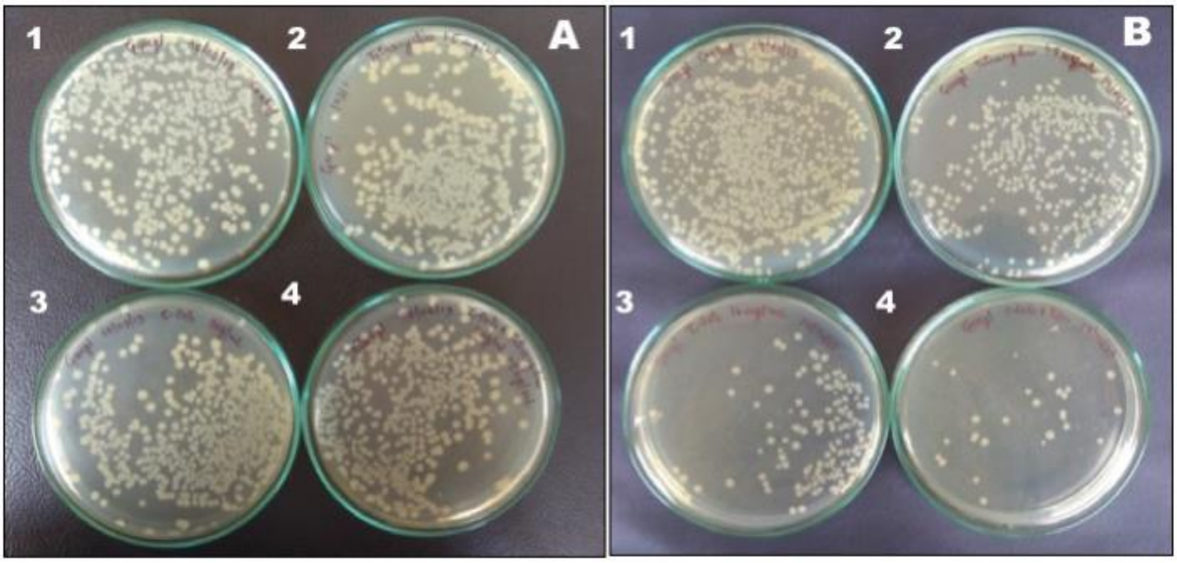

Suppl. Figure 4 | LB agar plates after synergistic treatment with tetracycline and C-dot. A) 1- Control, 2- tetracycline 1.5 µg/mL, 3- C-dot 8 µg/mL, 4- 1.5 µg/mL tetracycline + 8 µg/mL C-dot. B) 1- Control, 2- tetracycline 1.5 µg/mL, 3- C-dot 16 µg/mL, 4- 1.5 µg/mL tetracycline + 16 µg/mL C-dot.

(a)

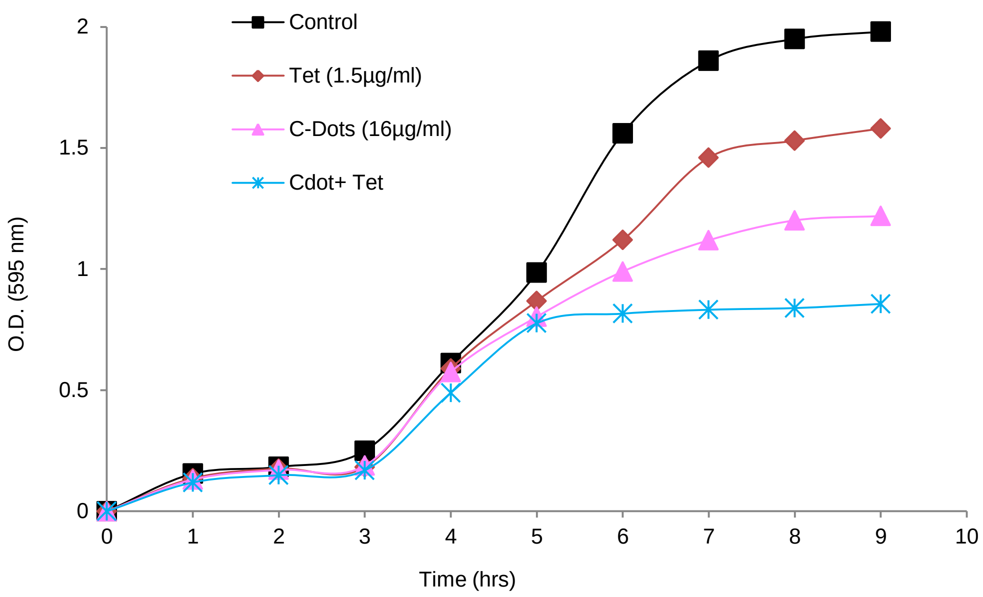

(b)

```
(*
data
 time, od,    od1,    od2, od3
 0,      0,       0,     0, 0
 1, 0.155, 0.136, 0.131, 0.119
 2, 0.183, 0.176, 0.171, 0.149
 3, 0.25, 0.182, 0.188,  0.169
 4, 0.612,0.589, 0.575,  0.489
 5, 0.985, 0.868, 0.803, 0.776
 6, 1.56,  1.12, 0.99,   0.816
 7, 1.86,  1.46, 1.119,  0.832
 8, 1.95,  1.53, 1.201,  0.839
 9, 1.98,  1.58, 1.219,  0.856
*)
(*
Zwietering,M.H.,et al."Modeling of the bacterial growth curve." Applied and environmental microbiology 56.6 (1990):1875-1881.
*)
datx = {{0,   0,    0,   0, 0}, {1, 0.155, 0.136, 0.131, 0.119}, {2, 0.183, 0.176, 0.171, 0.149}, {3, 0.25, 0.182, 0.188, 0.169},
   {4, 0.612, 0.589, 0.575, 0.489}, {5, 0.985, 0.868, 0.803, 0.776}, {6, 1.56, 1.12, 0.99, 0.816}, {7, 1.86, 1.46, 1.119, 0.832},
   {8, 1.95, 1.53, 1.201, 0.839}, {9, 1.98`, 1.58, 1.219, 0.856}};
```

$$\text{logistic} = \frac{A}{1 + \mathrm{Exp}\left[\frac{4\,\mu_m}{A}\,(\lambda - t) + 2\right]};$$

$$\text{gompertz} = A\,\mathrm{Exp}\left[-\mathrm{Exp}\left[\frac{\mu_m\,\mathrm{Exp}[1]}{A}\,(\lambda - t) + 1\right]\right];$$

$$\text{richards} = A\left(1 + \nu\,\mathrm{Exp}[1 + \nu]\,\mathrm{Exp}\left[\frac{\mu_m}{A}\,(1 + \nu)\left(1 + \frac{1}{\nu}\right)(\lambda - t)\right]\right)^{\frac{-1}{\nu}};$$

$$\text{stannard} = A\left(1 + \nu\,\mathrm{Exp}[1 + \nu]\,\mathrm{Exp}\left[\frac{\mu_m}{A}\,(1 + \nu)\left(1 + \frac{1}{\nu}\right)(\lambda - t)\right]\right)^{\frac{-1}{\nu}};$$

1. Control

```
(*Control *)
logfitc = FindFit[datx[[1 ;;, {1, 2}]], logistic, {A, μm, λ}, t]
gomfitc = FindFit[datx[[1 ;;, {1, 2}]], gompertz, {A, μm, λ}, t]
richardsfitc = FindFit[datx[[1 ;;, {1, 2}]], richards, {A, μm, λ, ν}, t]
stannardfitc = FindFit[datx[[1 ;;, {1, 2}]], stannard, {A, μm, λ, ν}, t]
Show[ListPlot[datx[[1 ;;, {1, 2}]], PlotStyle → Directive[PointSize[0.03], PlotLegends → Placed[{"Group"}, {0.28, 0.9}], Black],
  AxesLabel → {Style["time  (in hr)", Black, FontFamily → "Zapfino", FontSize → 18],
   Style["Optical  Density-Control", Black, FontFamily → "Zapfino", FontSize → 18]}],
 Show[Plot[Evaluate[logistic /. logfitc], {t, 0, 10}, PlotLegends → Placed["Expressions", {0.28, 0.9}], PlotStyle → {Thick, Dashed, Red}]],
 Show[Plot[Evaluate[gompertz /. gomfitc], {t, 0, 10}, PlotLegends → Placed["Expressions", {0.28, 0.8}],
   PlotStyle → {Thick, Dotted, Blue}]],
 Show[Plot[Evaluate[richards /. richardsfitc], {t, 0, 10}, PlotLegends → Placed["Expressions", {0.28, 0.7}],
   PlotStyle → {Thick, Dashed, Green}]],
 Show[Plot[Evaluate[stannard /. stannardfitc], {t, 0, 10},  PlotLegends → Placed["Expressions", {0.28, 0.6}],
   PlotStyle → {Thick, Dashed, Black}]], ImageSize → 800]
```

$\{A \to 2.06178,\ \mu_m \to 0.492999,\ \lambda \to 2.84817\}$

$\{A \to 2.20274,\ \mu_m \to 0.468312,\ \lambda \to 2.65181\}$

$\{A \to 1.97522,\ \mu_m \to 0.668283,\ \lambda \to 3.21903,\ \nu \to 2.88555\}$

$\{A \to 1.97522,\ \mu_m \to 0.668283,\ \lambda \to 3.21903,\ \nu \to 2.88555\}$

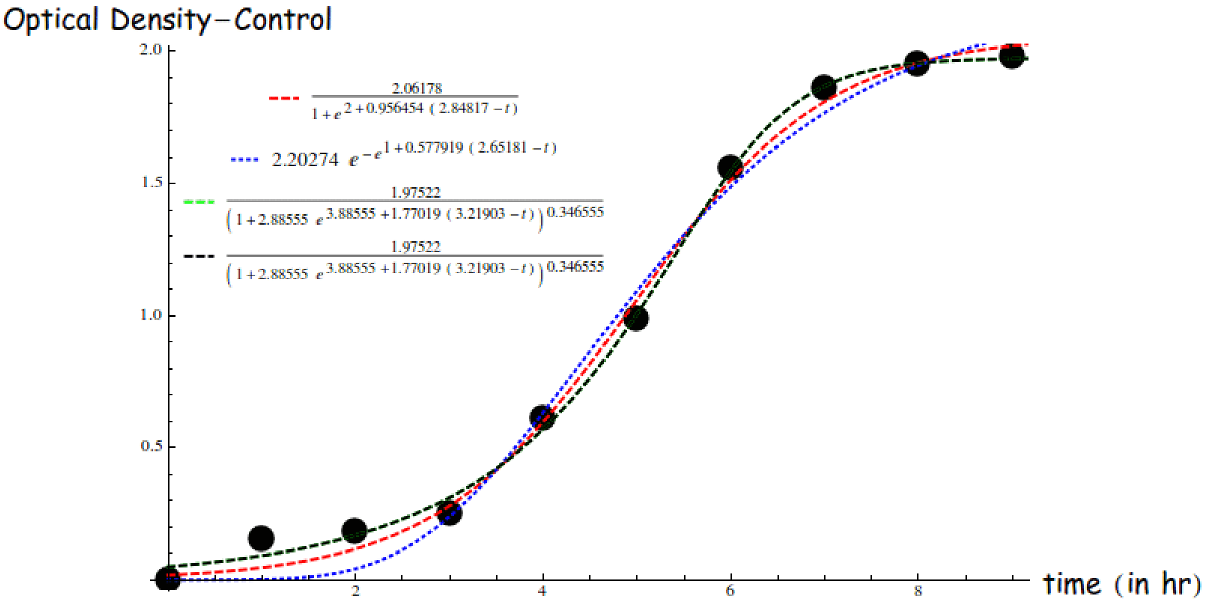

2. Tetracycline

```
logfita = FindFit[datx[[1 ;;, {1, 3}]], logistic, {A, μm, λ}, t]
gomfita = FindFit[datx[[1 ;;, {1, 3}]], gompertz, {A, μm, λ}, t]
richardsfita = FindFit[datx[[1 ;;, {1, 3}]], richards, {A, μm, λ, ν}, t]
stannardfita = FindFit[datx[[1 ;;, {1, 3}]], stannard, {A, μm, λ, ν}, t]
Show[ListPlot[datx[[1 ;;, {1, 3}]], PlotStyle → Directive[PointSize[0.03], PlotLegends → Placed[{"Group"}, {0.28, 0.9}], Black],
  AxesLabel → {Style["time (in hr)", Black, FontFamily → "Zapfino", FontSize → 18],
    Style["Optical Density-Tet", Black, FontFamily → "Zapfino", FontSize → 18]}],
 Show[Plot[Evaluate[logistic /. logfita], {t, 0, 10}, PlotLegends → Placed["Expressions", {0.28, 0.9}], PlotStyle → {Thick, Dashed, Red}]],
 Show[Plot[Evaluate[gompertz /. gomfita], {t, 0, 10}, PlotLegends → Placed["Expressions", {0.28, 0.8}],
   PlotStyle → {Thick, Dotted, Blue}]],
 Show[Plot[Evaluate[richards /. richardsfita], {t, 0, 10}, PlotLegends → Placed["Expressions", {0.28, 0.7}],
   PlotStyle → {Thick, Dashed, Green}]],
 Show[Plot[Evaluate[stannard /. stannardfita], {t, 0, 10}, PlotLegends → Placed["Expressions", {0.28, 0.6}],
   PlotStyle → {Thick, Dashed, Black}]], ImageSize → 800]
```

{A → 1.65079, μ_m → 0.340886, λ → 2.47841}
{A → 1.8103, μ_m → 0.318273, λ → 2.22747}
{A → 1.62945, μ_m → 0.371258, λ → 2.54647, ν → 1.27521}
{A → 1.62945, μ_m → 0.371258, λ → 2.54647, ν → 1.27521}

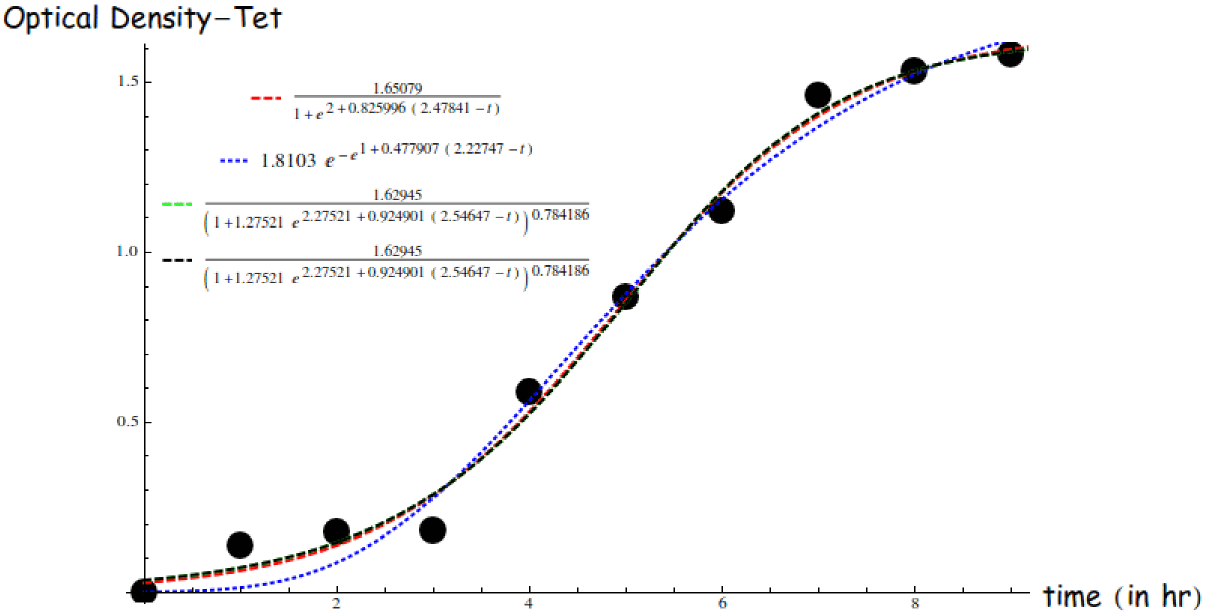

3. C-dot

```
(*Cdots*)
logfitb = FindFit[datx[[1 ;;, {1, 4}]], logistic, {A, μm, λ}, t]
gomfitb = FindFit[datx[[1 ;;, {1, 4}]], gompertz, {A, μm, λ}, t]
richardsfitb = FindFit[datx[[1 ;;, {1, 4}]], richards, {A, μm, λ, ν}, t]
stannardfitb = FindFit[datx[[1 ;;, {1, 4}]], stannard, {A, μm, λ, ν}, t]
Show[ListPlot[datx[[1 ;;, {1, 4}]], PlotStyle → Directive[PointSize[0.03], PlotLegends → Placed[{"Group"}, {0.28, 0.9}], Black],
  AxesLabel → {Style["time (in hr)", Black, FontFamily → "Zapfino", FontSize → 18],
    Style["Optical Density-Cdots", Black, FontFamily → "Zapfino", FontSize → 18]}],
 Show[Plot[Evaluate[logistic /. logfitb], {t, 0, 10}, PlotLegends → Placed["Expressions", {0.28, 0.9}], PlotStyle → {Thick, Dashed, Red}]],
 Show[Plot[Evaluate[gompertz /. gomfitb], {t, 0, 10}, PlotLegends → Placed["Expressions", {0.28, 0.8}],
   PlotStyle → {Thick, Dotted, Blue}]],
 Show[Plot[Evaluate[richards /. richardsfitb], {t, 0, 10}, PlotLegends → Placed["Expressions", {0.28, 0.7}],
   PlotStyle → {Thick, Dashed, Green}]],
 Show[Plot[Evaluate[stannard /. stannardfitb], {t, 0, 10}, PlotLegends → Placed["Expressions", {0.28, 0.6}],
   PlotStyle → {Thick, Dashed, Black}]], ImageSize → 800]
```

$\{A \to 1.23715,\ \mu_m \to 0.274339,\ \lambda \to 2.09061\}$

$\{A \to 1.32668,\ \mu_m \to 0.25837,\ \lambda \to 1.87111\}$

$\{A \to 1.2295,\ \mu_m \to 0.289259,\ \lambda \to 2.12538,\ \nu \to 1.1613\}$

$\{A \to 1.2295,\ \mu_m \to 0.289259,\ \lambda \to 2.12538,\ \nu \to 1.1613\}$

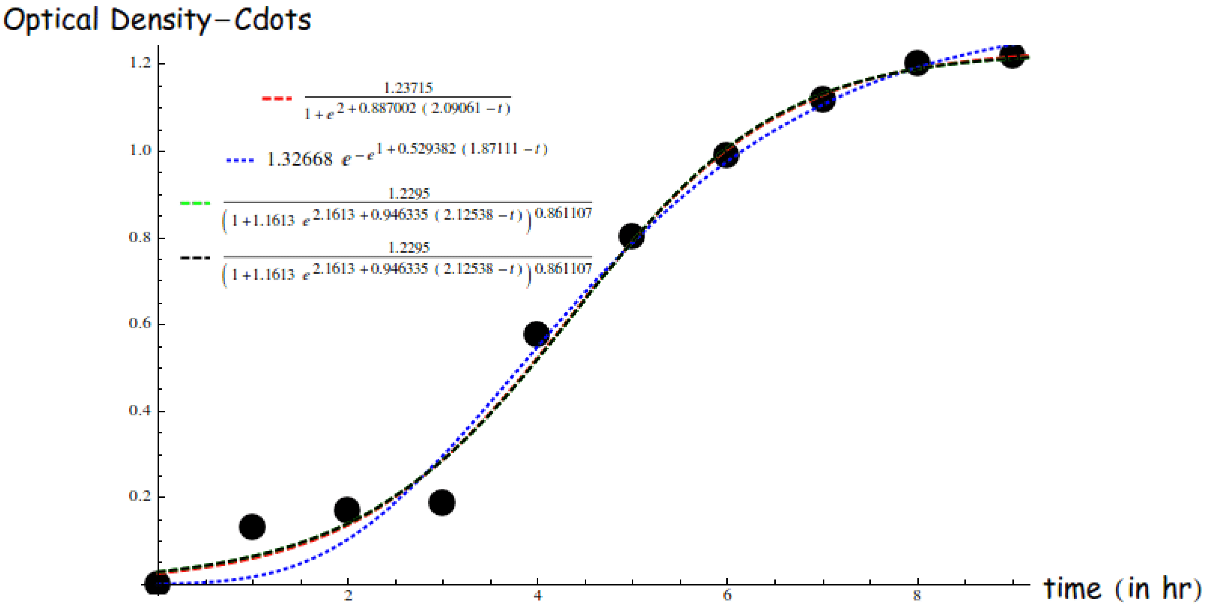

4. C-dot + Tetracycline

```
(*Cdots + tet *)
logfitd = FindFit[datx[[1 ;;, {1, 5}]], logistic, {A, μm, λ}, t]
gomfitd = FindFit[datx[[1 ;;, {1, 5}]], gompertz, {A, μm, λ}, t]
richardsfitd = FindFit[datx[[1 ;;, {1, 5}]], {richards, μm > 0, λ > 2, ν > 1}, {A, μm, λ, ν}, t]
stannardfitd = FindFit[datx[[1 ;;, {1, 5}]], {stannard, μm > 0, λ > 2, ν > 1}, {A, μm, λ, ν}, t]
Show[ListPlot[datx[[1 ;;, {1, 5}]], PlotStyle → Directive[PointSize[0.03], PlotLegends → Placed[{"Group"}, {0.28, 0.9}], Black],
  AxesLabel → {Style["time (in hr)", Black, FontFamily → "Zapfino", FontSize → 18],
    Style["Optical Density-Cdots+Tet", Black, FontFamily → "Zapfino", FontSize → 18]}],
 Show[Plot[Evaluate[logistic /. logfitd], {t, 0, 10}, PlotLegends → Placed["Expressions", {0.28, 0.9}], PlotStyle → {Thick, Dashed, Red}]],
 Show[Plot[Evaluate[gompertz /. gomfitd], {t, 0, 10}, PlotLegends → Placed["Expressions", {0.28, 0.8}],
   PlotStyle → {Thick, Dotted, Blue}]],
 Show[Plot[Evaluate[richards /. richardsfitd], {t, 0, 10}, PlotLegends → Placed["Expressions", {0.28, 0.7}],
   PlotStyle → {Thick, Dashed, Green}]],
 Show[Plot[Evaluate[stannard /. stannardfitd], {t, 0, 10}, PlotLegends → Placed["Expressions", {0.28, 0.6}],
   PlotStyle → {Thick, Dashed, Black}]], ImageSize → 800]
```

```
{A → 0.857394, μm → 0.285429, λ → 2.24359}
{A → 0.862259, μm → 0.336314, λ → 2.43422}
{A → 0.83611, μm → 0.418906, λ → 2.6877, ν → 5.56846}
{A → 0.83611, μm → 0.418906, λ → 2.6877, ν → 5.56846}
```

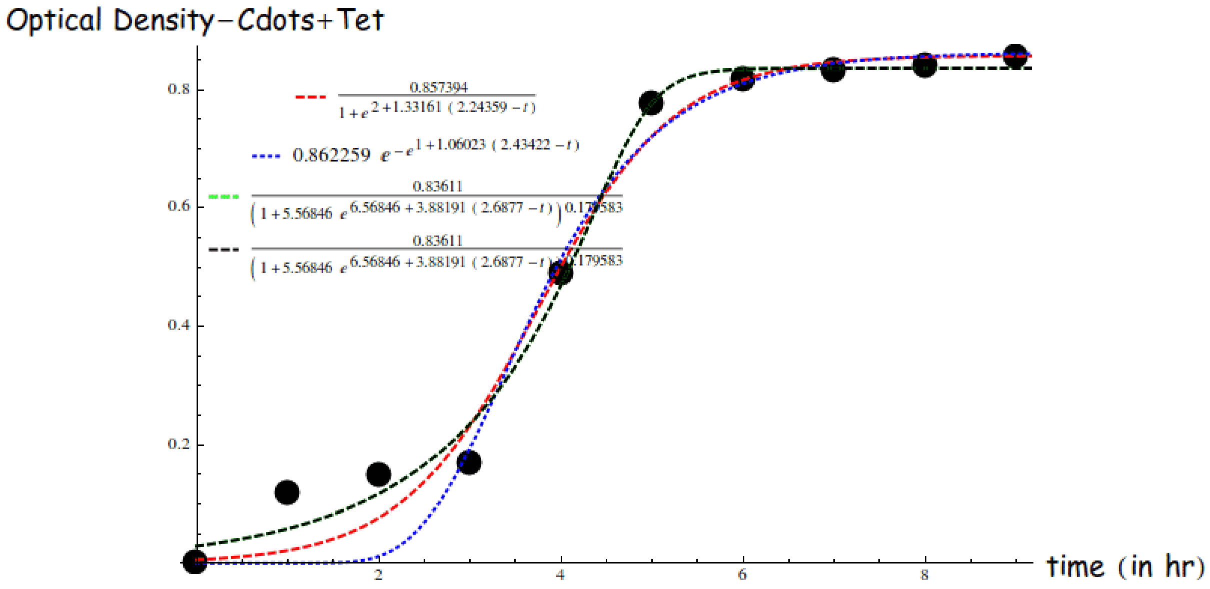

Suppl. Figure 5 | Growth curve of *E. coli* and Mathematica script for fitting functions (a) Growth curve treated with tetracycline, C-dot and synergistically with both and (b) Mathematica script for fitting functions of logistic (1), Gompartz (2), Richards (3) and Stannard (4) models (Table 1 is given for detail fitting parameters).

(a)

a

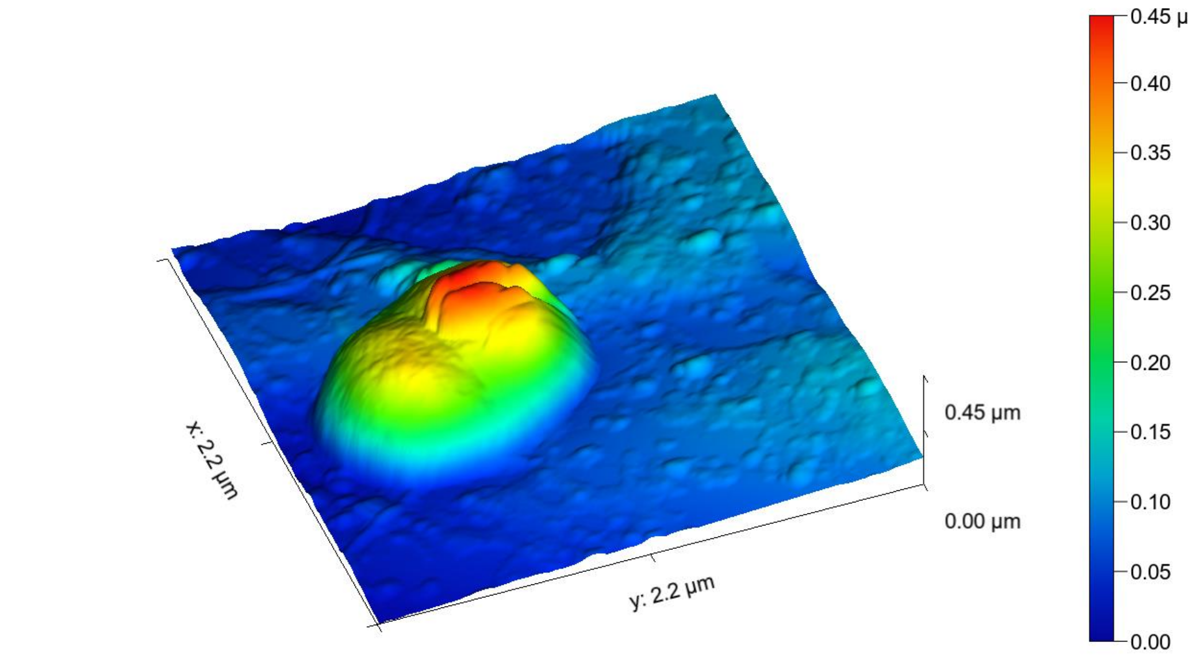

0.45 µ
0.40
0.35
0.30
0.25
0.20
0.15
0.10
0.05
0.00
0.45 µm
0.00 µm
x: 2.2 µm
y: 2.2 µm

b

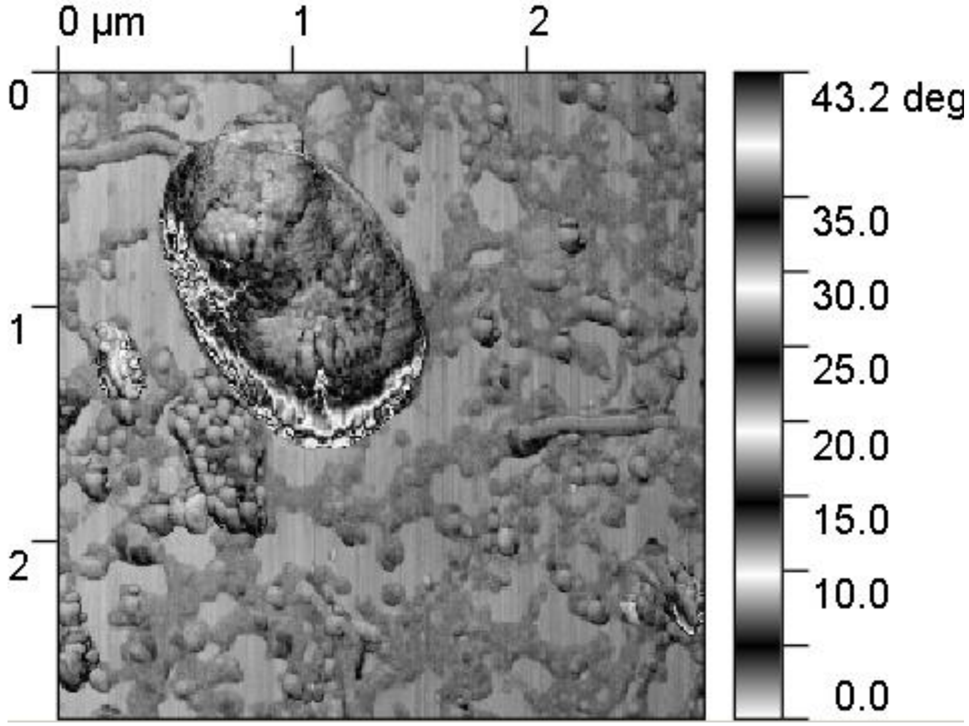

0 µm
1
2
0
1
2
43.2 deg
35.0
30.0
25.0
20.0
15.0
10.0
0.0

c

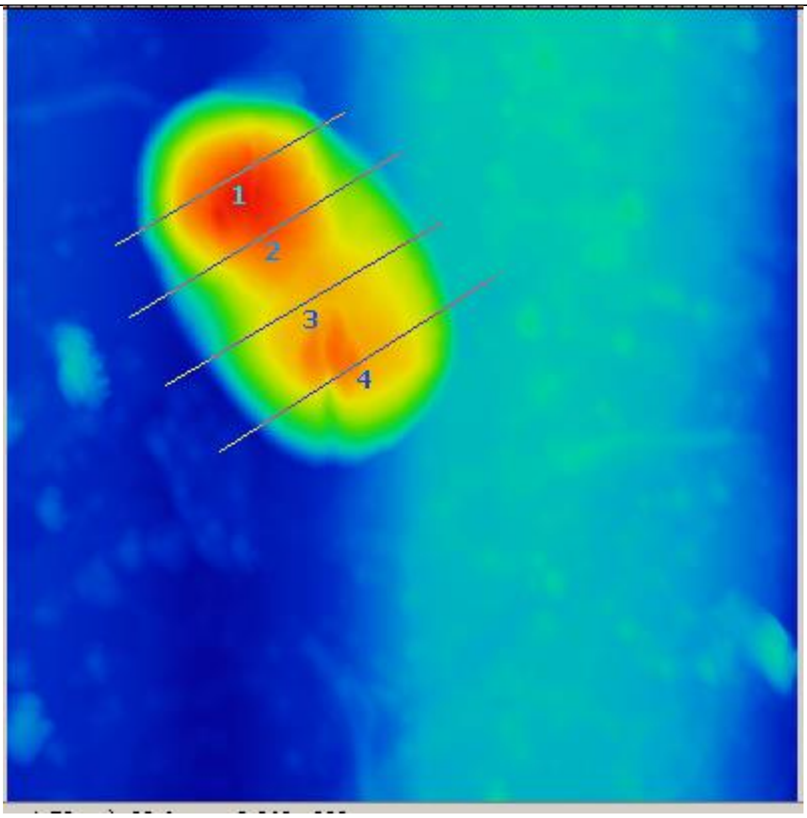
1
2
3
4

d

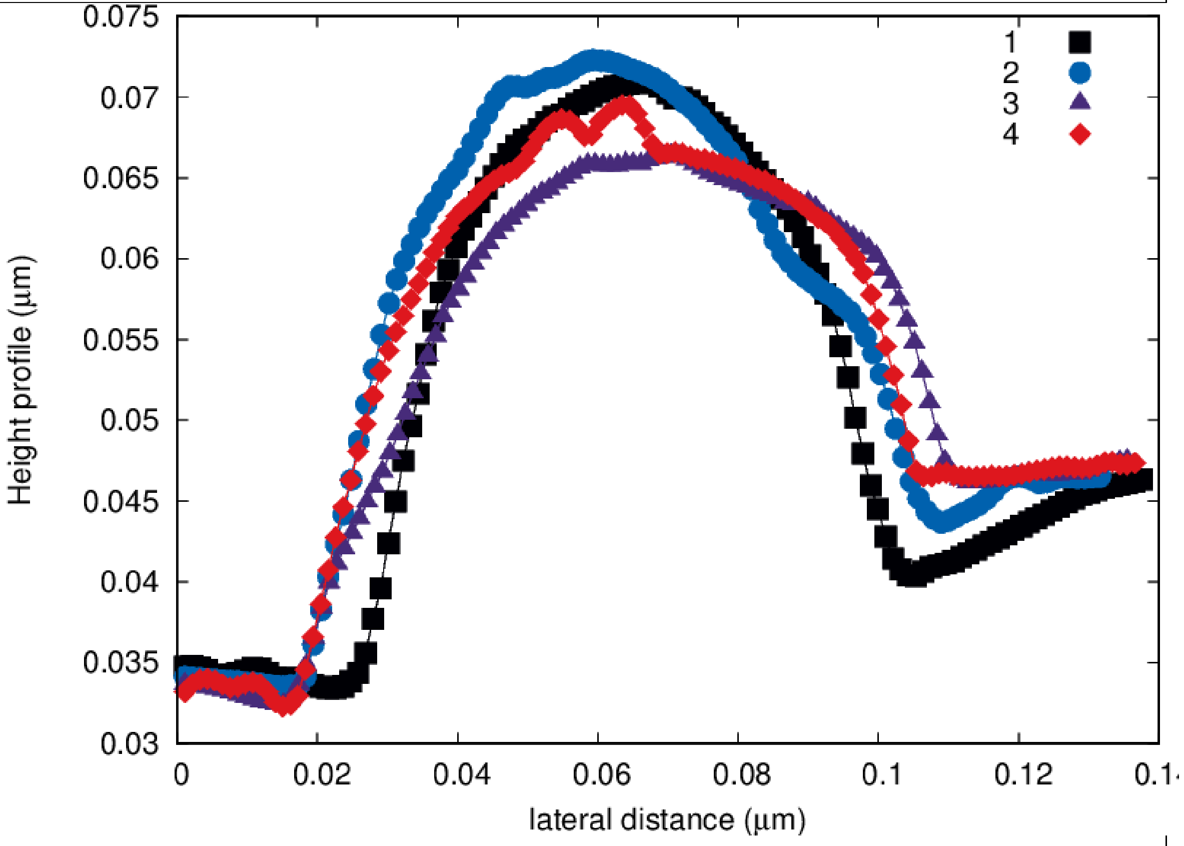
0.075
0.07
0.065
0.06
0.055
0.05
0.045
0.04
0.035
0.03
Height profile (µm)
0
0.02
0.04
0.06
0.08
0.1
0.12
lateral distance (µm)
1
2
3
4

**e**	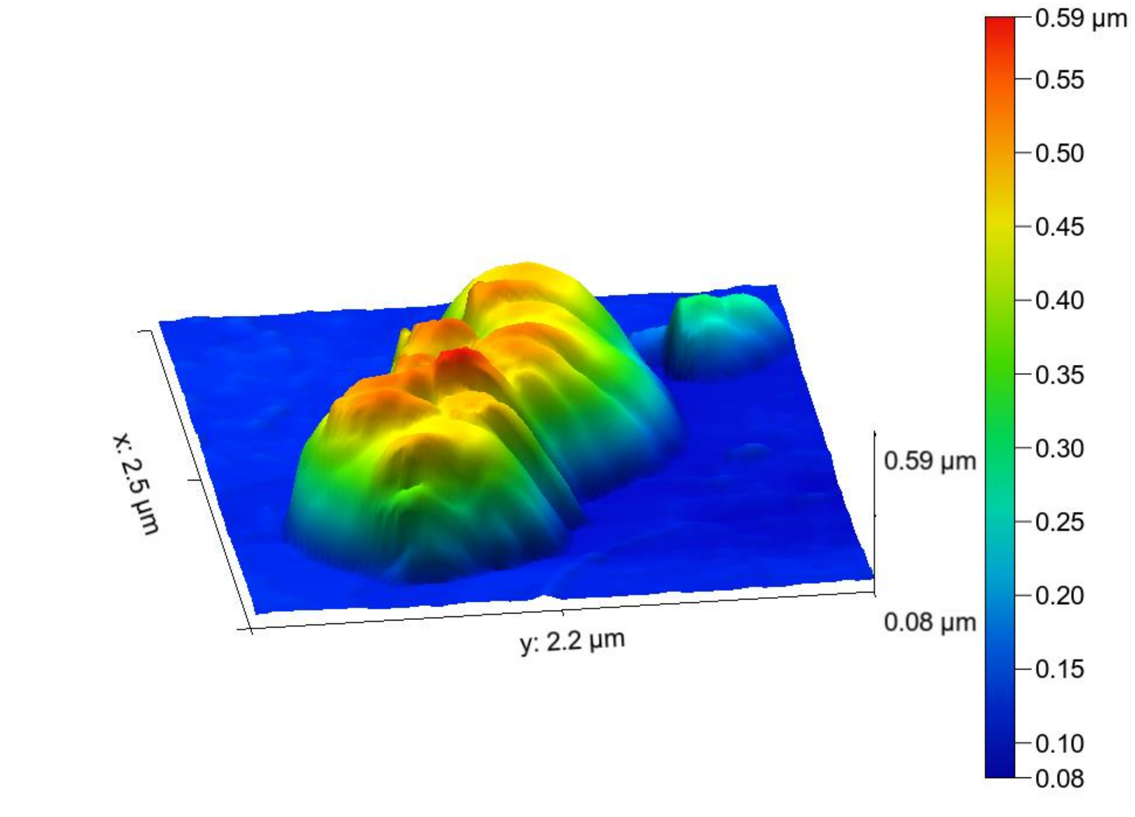 0.59 µm<br>0.55<br>0.50<br>0.45<br>0.40<br>0.35<br>0.30<br>0.25<br>0.20<br>0.15<br>0.10<br>0.08<br>x: 2.5 µm<br>y: 2.2 µm<br>0.59 µm<br>0.08 µm
**f**	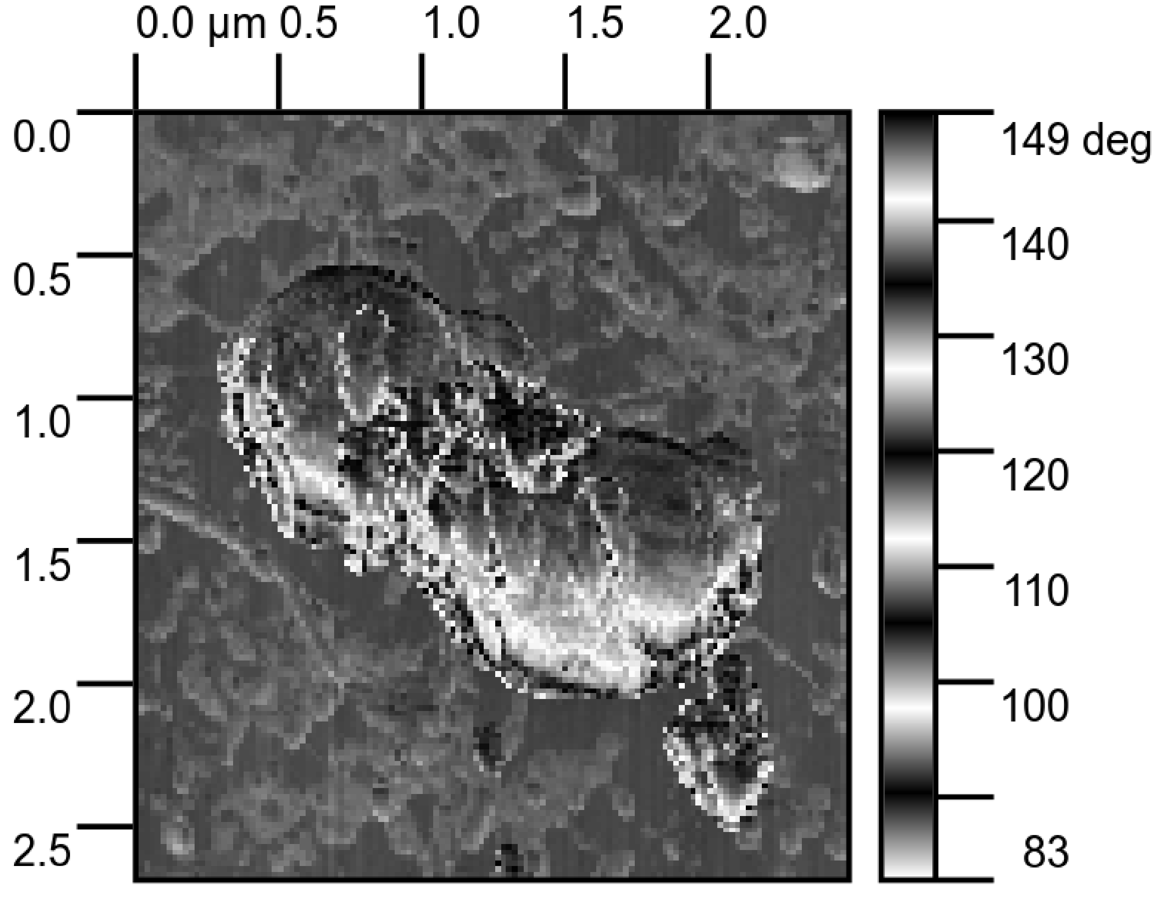 0.0 µm 0.5<br>1.0<br>1.5<br>2.0<br>0.0<br>0.5<br>1.0<br>1.5<br>2.0<br>2.5<br>149 deg<br>140<br>130<br>120<br>110<br>100<br>83

g	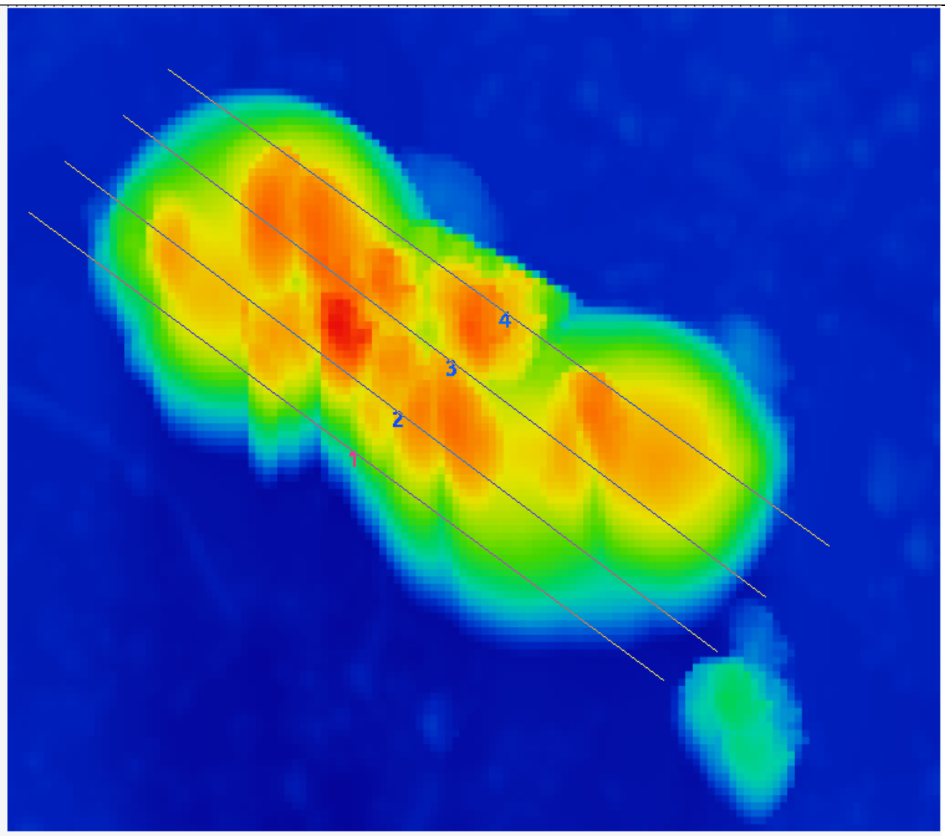1<br>2<br>3<br>4
h	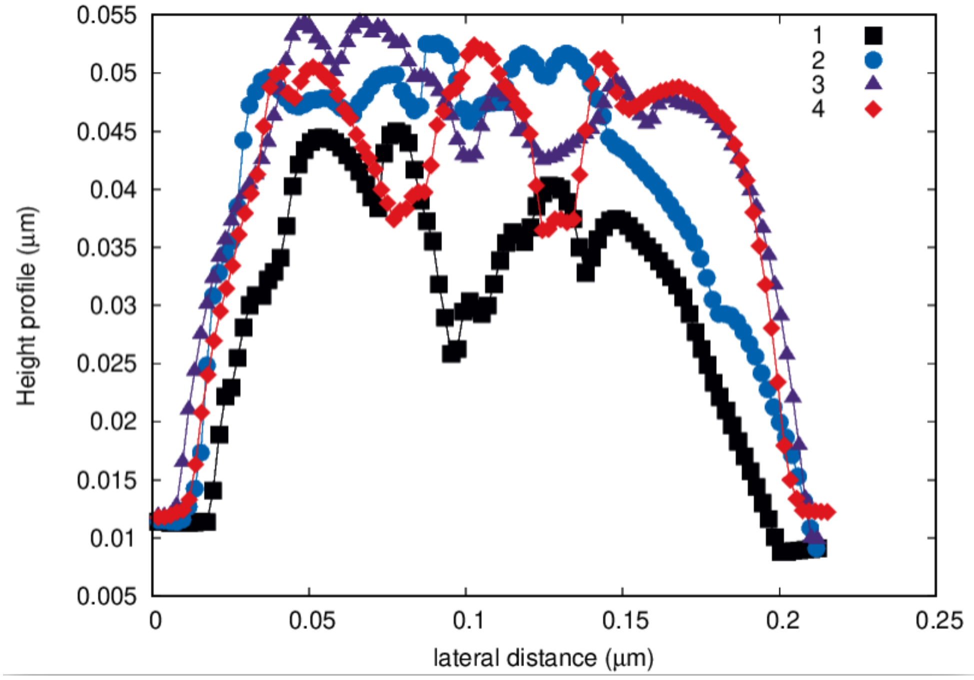Height profile (µm)<br>lateral distance (µm)<br>0.055<br>0.05<br>0.045<br>0.04<br>0.035<br>0.03<br>0.025<br>0.02<br>0.015<br>0.01<br>0.005<br>0<br>0.05<br>0.1<br>0.15<br>0.2<br>0.25<br>1<br>2<br>3<br>4

i

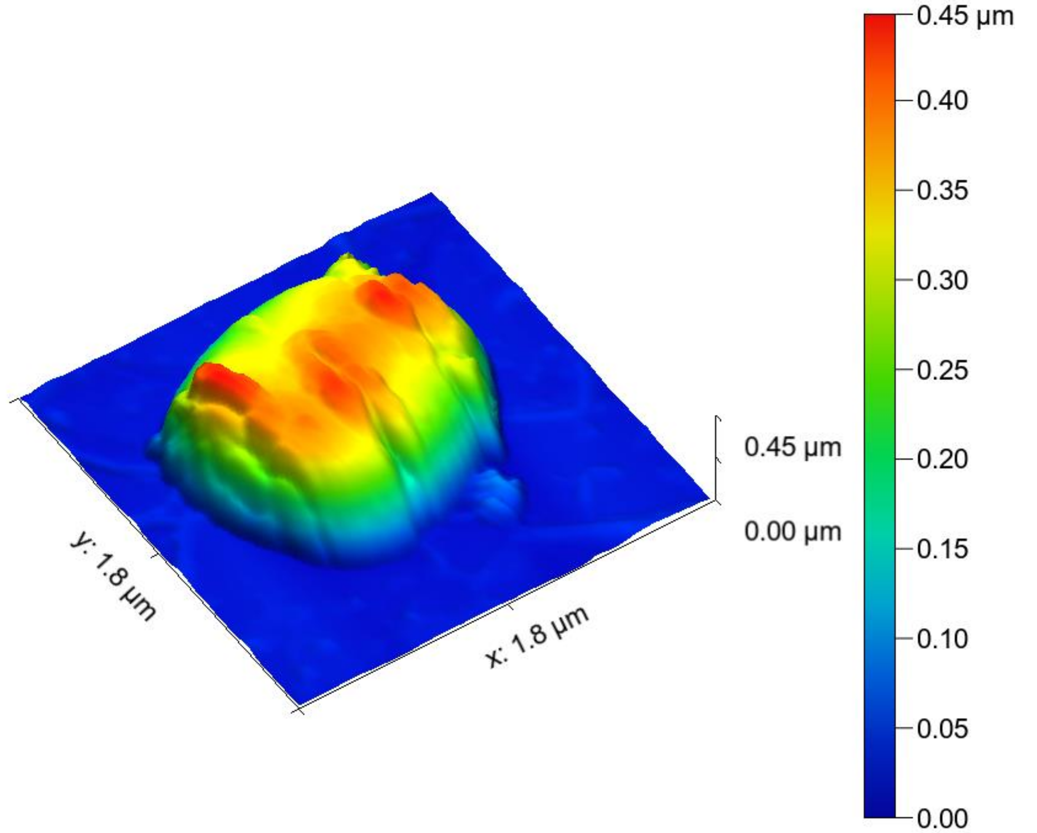
0.45 µm
0.40
0.35
0.30
0.25
0.20
0.15
0.10
0.05
0.00
0.45 µm
0.00 µm
y: 1.8 µm
x: 1.8 µm

j

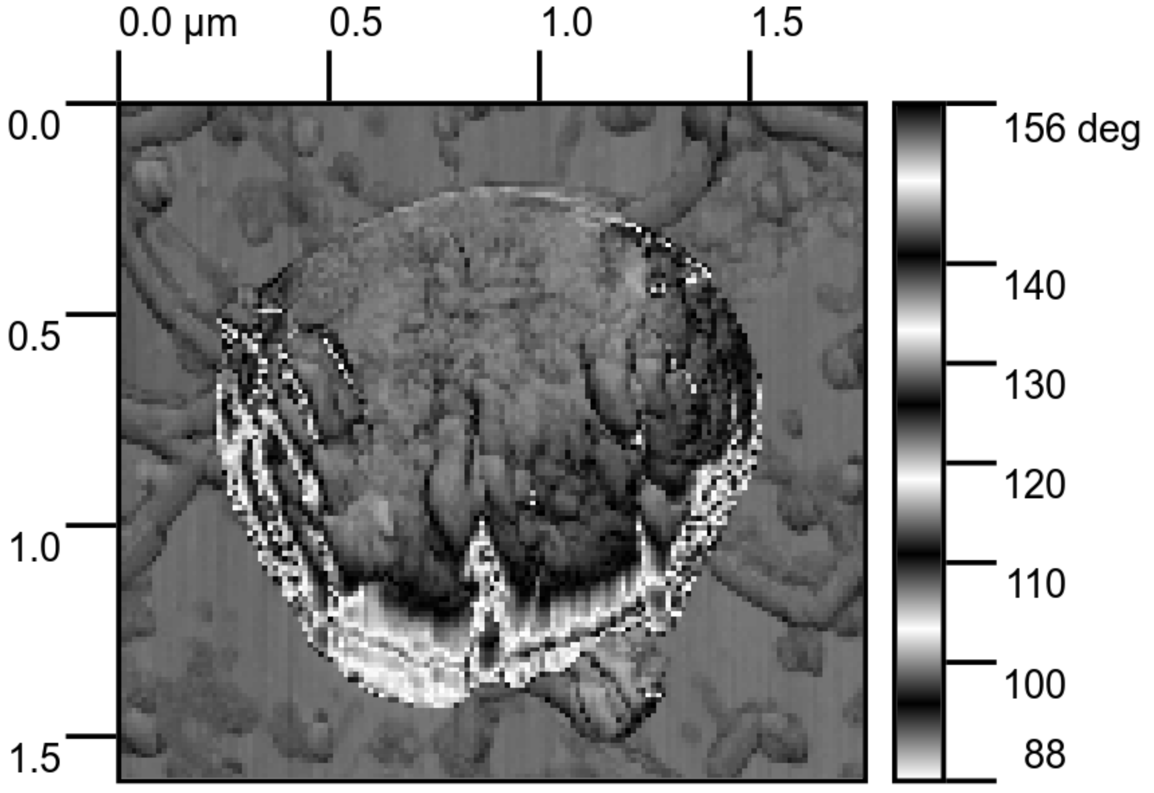
0.0 µm
0.5
1.0
1.5
0.0
0.5
1.0
1.5
156 deg
140
130
120
110
100
88

**k**	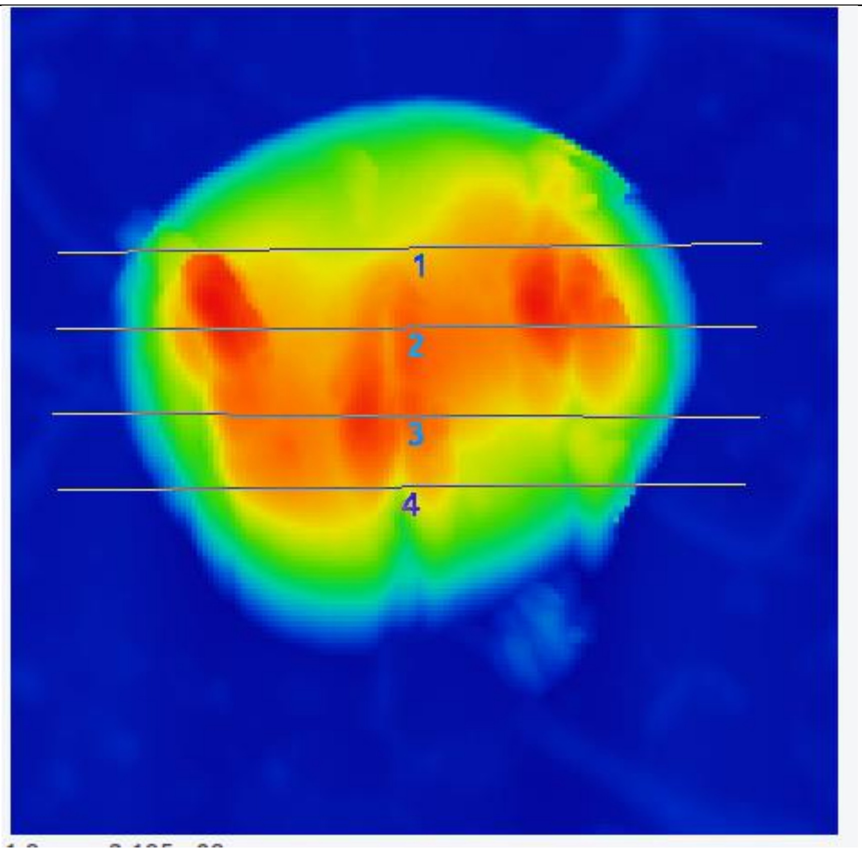1<br>2<br>3<br>4
**l**	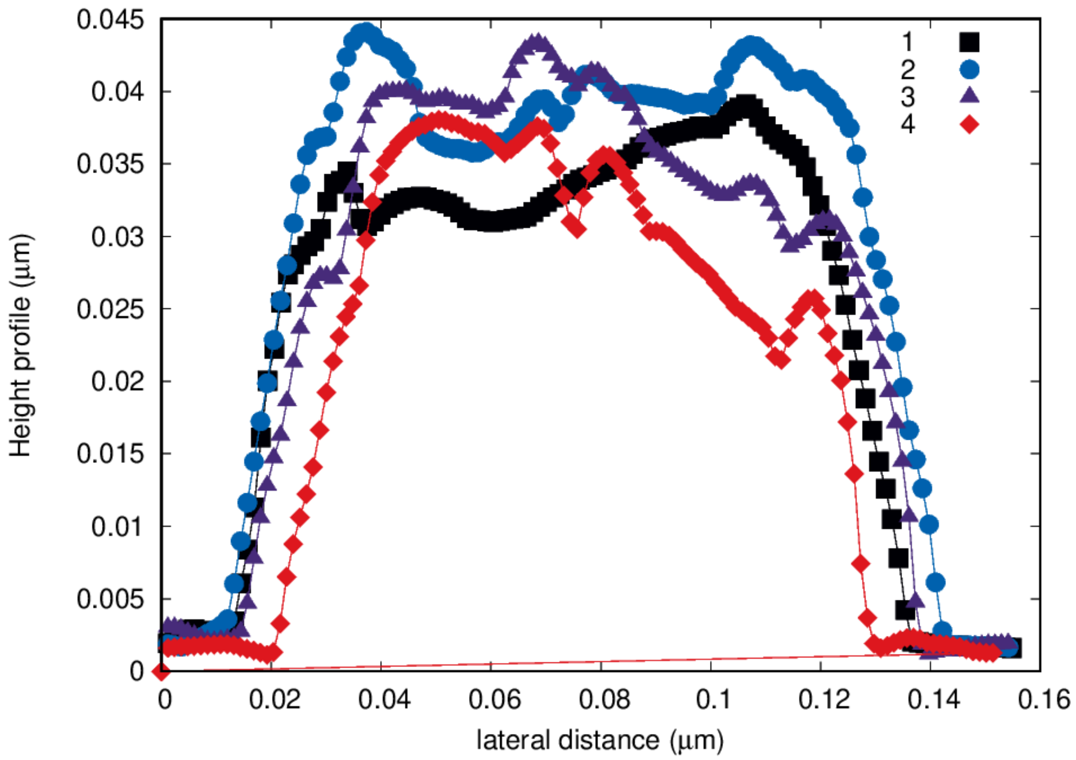Height profile (µm)<br>lateral distance (µm)<br>0.045<br>0.04<br>0.035<br>0.03<br>0.025<br>0.02<br>0.015<br>0.01<br>0.005<br>0<br>0.02<br>0.04<br>0.06<br>0.08<br>0.1<br>0.12<br>0.14<br>0.16<br>1<br>2<br>3<br>4

(b)

Control

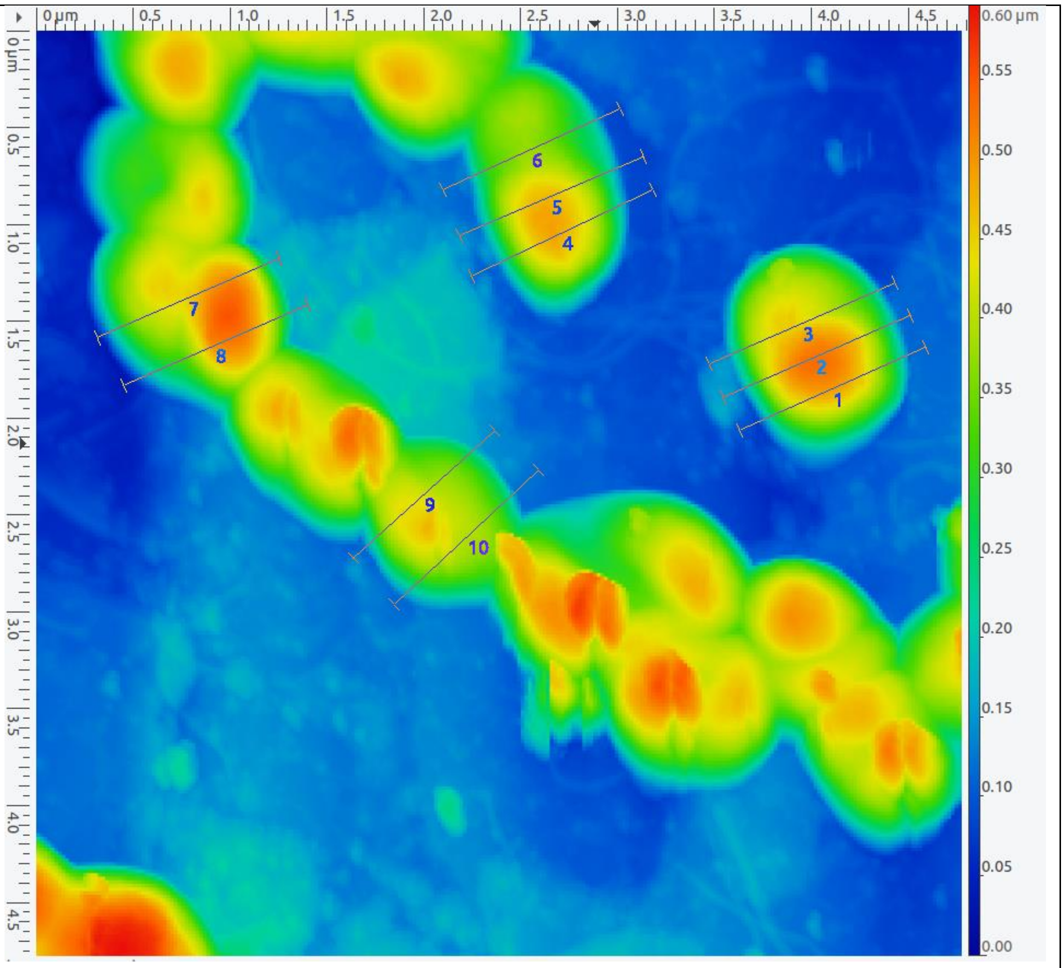

Control-graph

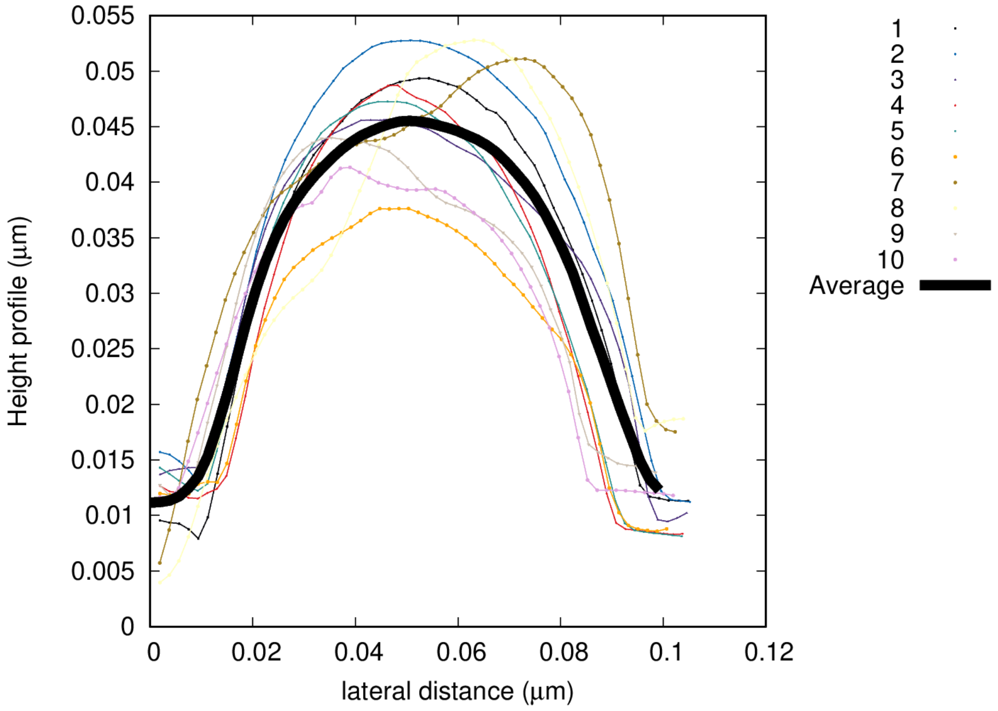

**C-dot**	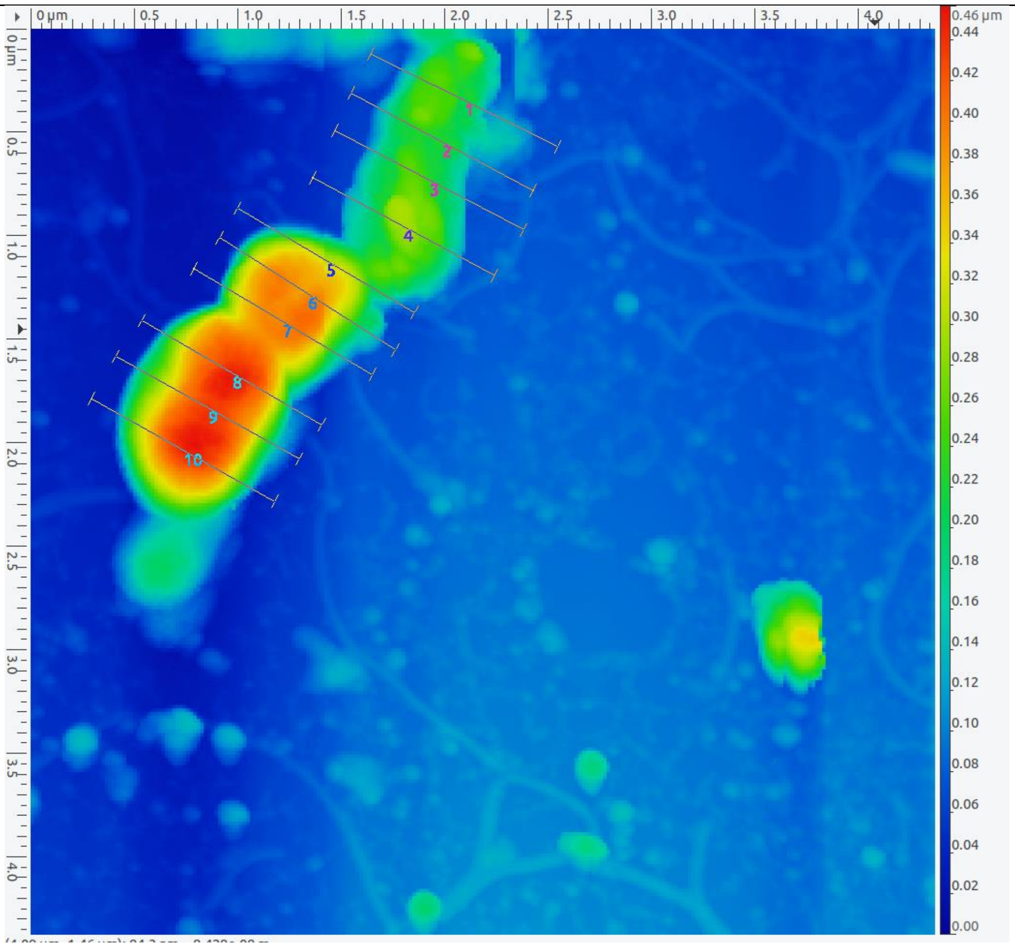
**C-dot-graph**	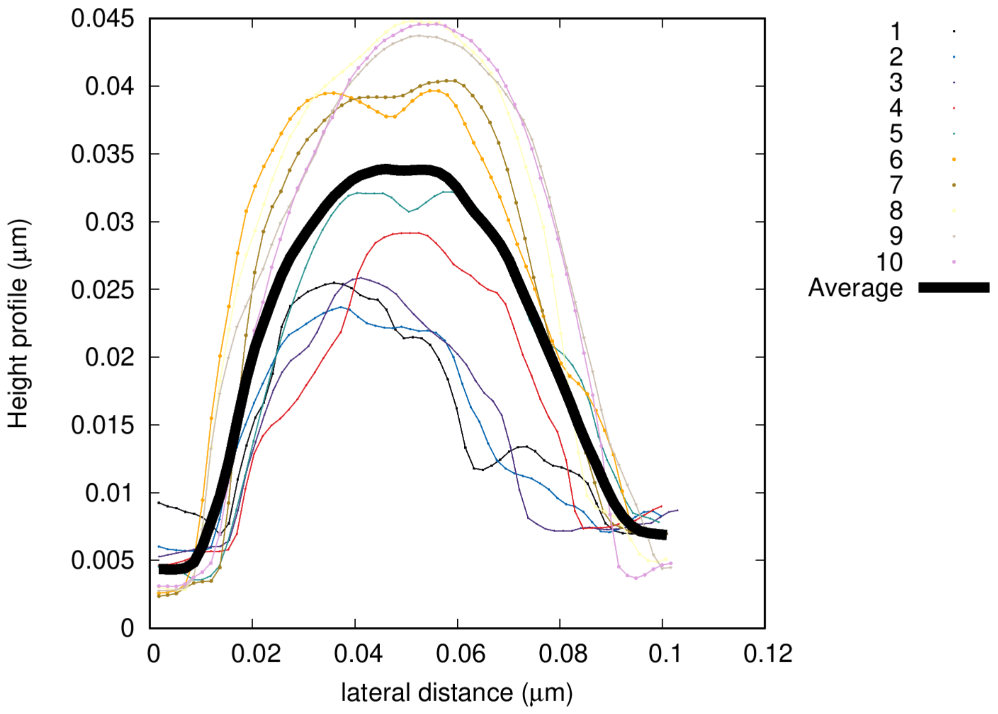

**C-**<br>**dot+tet**	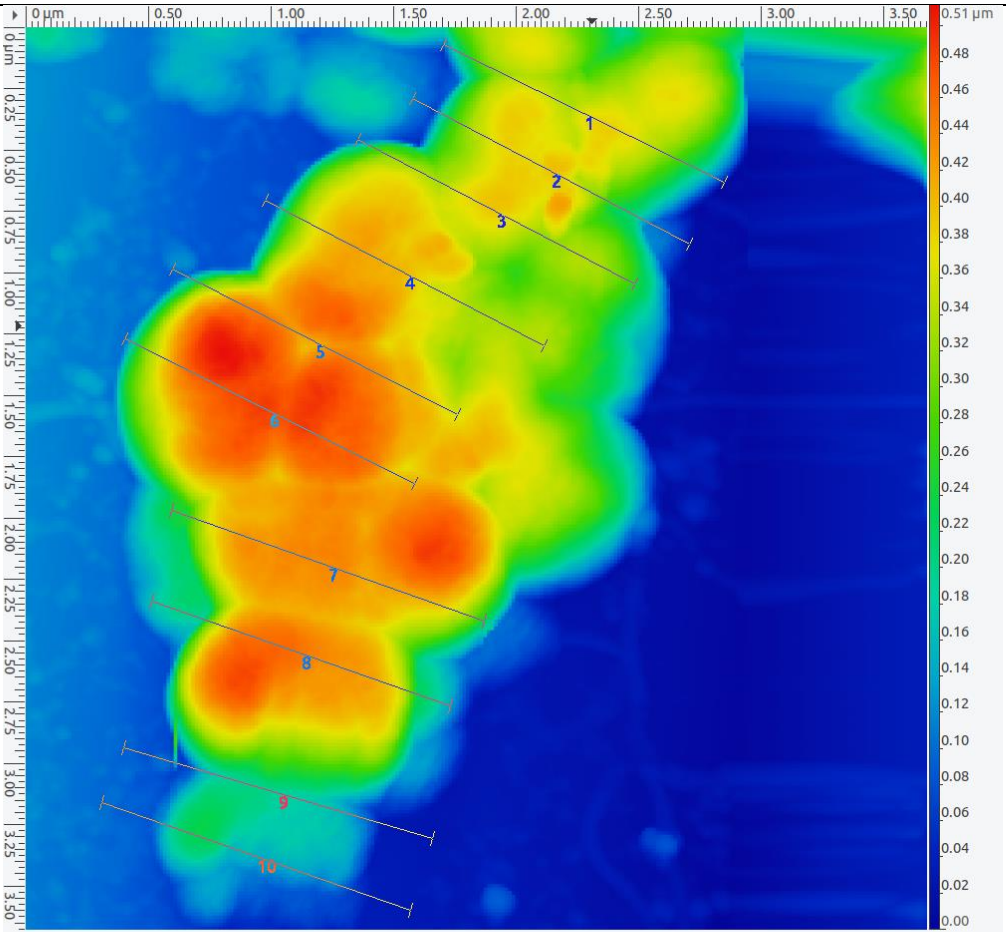
**C-**<br>**dot+tet-**<br>**graph**	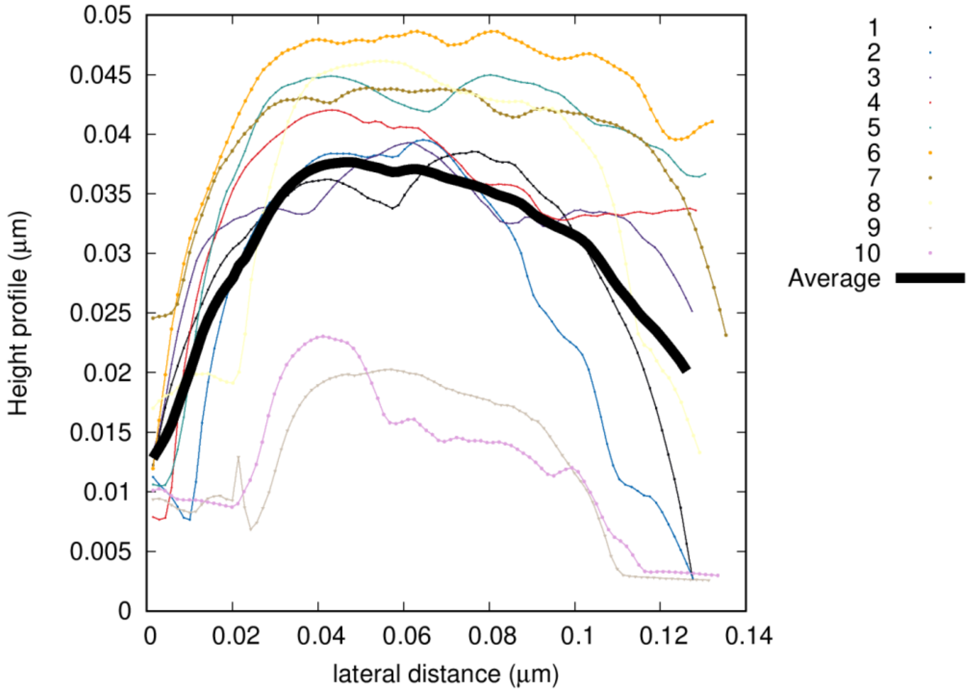

Suppl. Figure 6 | (a) High resolution AFM images of *E. coli* surface topography as described in Fig.5, **(b)** The line profile data average over multiple cells.

Suppl. Table 1 | Zone of inhibition assay of tetracycline.

Tetracycline concentrations(μg/mL)	Average Zone of inhibition (mm)
0.25	-
0.5	-
0.75	-
1.0	-
1.5	6
2.0	8
2.5	9
3.0	10
3.5	10
4.0	11
4.5	12
5.0	13
Control	-

Suppl. Table 2 | Observation table for CFU (C-dot treated *E. coli*).

C-dot (μg/mL)	No. of colonies	CFU/mL
Control	238.6±7	4.8×10^{7}±1418920
8	103.3±6	2.1×10^{7}±1222020
16	44.3±4	8.9×10^{6}±808290
32	35.3±3	7.1×10^{6}±642910
64	23.3±3	4.7×10^{6}±702376

Suppl. Table 3 | Observation table for CFU for study of synergistic effect of C-dot and tetracycline**.**

Concentration (µg/mL)	No. of Colonies	CFU/mL
Control	382±5.2	7.64×10^{7}±1058301
1.5 (tet)	275±8.8	5.5×10^{7}±1777639
8 (C-dot)	158±5	3.17×10^{7}±1006645
16 (C-dot)	78±6	1.57×10^{7}±1205543
C-dot (8 µg/mL + tet)	142±5.8	2.85×10^{7}±1171893
C-dot (16 µg/mL + tet)	37±5	7.4×10^{6}±1000000
